# Identifying Novel Transcriptional and Epigenetic Features of Nuclear Lamina-associated Genes

**DOI:** 10.1038/s41598-017-00176-x

**Published:** 2017-03-07

**Authors:** Feinan Wu, Jie Yao

**Affiliations:** Department of Cell Biology, Yale University School of Medicine, New Haven, CT 06520 USA

## Abstract

Because a large portion of the mammalian genome is associated with the nuclear lamina (NL), it is interesting to study how native genes resided there are transcribed and regulated. In this study, we report unique transcriptional and epigenetic features of nearly 3,500 NL-associated genes (NL genes). Promoter regions of active NL genes are often excluded from NL-association, suggesting that NL-promoter interactions may repress transcription. Active NL genes with higher RNA polymerase II (Pol II) recruitment levels tend to display Pol II promoter-proximal pausing, while Pol II recruitment and Pol II pausing are not correlated among non-NL genes. At the genome-wide scale, NL-association and H3K27me3 distinguishes two large gene classes with low transcriptional activities. Notably, NL-association is anti-correlated with both transcription and active histone mark levels among genes not significantly enriched with H3K9me3 or H3K27me3, suggesting that NL-association may represent a novel gene repression pathway. Interestingly, an NL gene subgroup is not significantly enriched with H3K9me3 or H3K27me3 and is transcribed at higher levels than the rest of NL genes. Furthermore, we identified distal enhancers associated with active NL genes and reported their epigenetic features.

## Introduction

The mammalian genome is compacted, spatially organized and compartmentalized within the cell nucleus^1^. The nuclear periphery represents an important subnuclear compartment for gene regulation^2, 3^. Genome-wide mapping has identified lamina associated domains (LADs) covering 30–40% of the mammalian genome^4–6^. Further analyses have identified different types of LADs according to their epigenetic compositions^7^, cell type-specific genome interactions^6^ or NL contact frequency among individual cells^8^. A large number of protein-coding genes reside in LADs^5^, but their transcriptional and epigenetic regulation are not well understood.

Several studies identified the connections between chromatin status, gene expression and gene localization at the nuclear periphery in mammalian cells. For example, conditional knockout of histone H3K9 methyltransferase G9a resulted in activation of a subset of genes located at the nuclear periphery in mouse embryonic stem cells^9^. Gene activation or chromatin decondensation induced with synthetic transcription factors resulted in repositioning of peripheral genes towards the nuclear interior^10^. Association of circadian genes with the NL in human cells was dependent upon PARP1 and CTCF and preceded their transcriptional repression^11^. While these case studies are highly informative, a genome-wide analysis will enable identifying transcriptional and epigenetic features of NL-associated genes that are critical for revealing their regulation.

We previously developed an approach to couple DNA adenine methyl-transferase Identification (DamID)^12^ to high throughput DNA sequencing (termed DamID-seq) that substantially improved the resolution and sensitivity of detecting NL interactions within genic regions^13, 14^. In this study, we identified a large number of NL genes, which are enriched in cell communication, signalling, ion transport, biological adhesion, etc. We used nascent RNA sequencing as a highly sensitive approach^15, 16^ to identify active NL genes and detect their unique transcriptional features. By genome-wide clustering analysis, we found that nuclear lamina association (NLA) and H3K27me3 are the predominant epigenetic features of two distinct classes of repressed genes. We found that NLA was anti-correlated with active histone marks among genes transcribed at the same level, indicating that active histone marks may be repressed near the NL. Furthermore, we identified NL gene subgroups that are differentially enriched with repressive histone marks and identified distal enhancers associated with active NL genes.

## Results

### NL genes identified by DamID-seq

To start this study, we identified NL genes using DamID-seq data on genome-NL interactions^14^ and mouse RefSeq genes^17^. Using high throughput sequencing data from cells expressing Dam-Lamin B1 fusion proteins (Dam-LmnB1) and cells expressing free Dam proteins (Dam-only)^14^, we defined NLA of a genomic region as log2 of read density ratio (Dam-LmnB1/Dam-only), where the read density was normalized to reads per kilo base per million (RPKM)^14^. We observed that expressed genes have lower NLA at regions proximal to transcription start sites (TSS) than silent genes^14^. Therefore, we defined NL genes as those genes having gene body (from TSS + 1 Kb to TES, i.e. transcript end site) NLA of 1 or higher and defined non-NL genes as those genes with gene body NLA less than −1. As a result, we identified 3,395 NL genes/15,712 non-NL genes in C2C12 myoblasts and 3,501 NL genes/16,473 non-NL genes in NIH 3T3 fibroblasts (Supplementary Tables S1 and S2). We further validated our approach of identifying NL genes by the fact that in NIH 3T3 fibroblasts, the majority (79.8%) of NL genes, in contrast to only 0.46% of non-NL genes, have their gene bodies overlapped with published LADs^5^ (Supplementary Table S3).

### A subset of NL genes is actively transcribed

**Figure 1:**
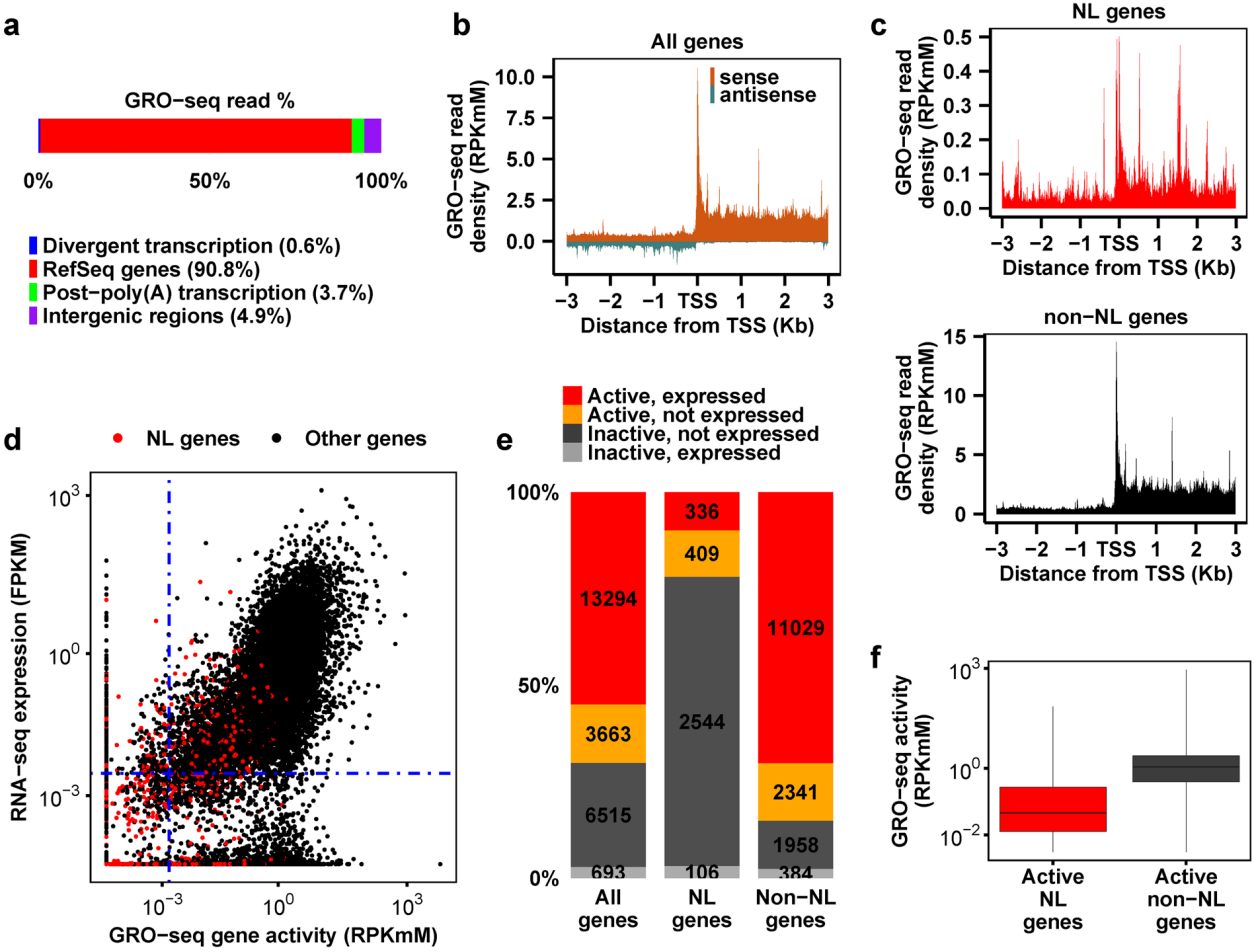
Genome-wide transcriptional activities measured by GRO-seq in C2C12 myoblasts. (**a**) The genome-wide distribution of GRO-seq reads. In total, 122 million GRO-seq reads were obtained from two biological replicates (Spearman’s ρ = 0.75 based on reads counts in 1 kb non-overlapping contiguous windows along the genome) and pooled for analyses. Reads in the 10 kb region downstream from the annotated TES on the same strand correspond to post-poly(A) transcription (green). Reads in the 5 kb region upstream from the annotated TSS on the opposite strand correspond to divergent transcription (blue). (**b**,**c**) Average GRO-seq read density in 10 bp bins along both sense and antisense strands among all genes (**b**) or along sense strands among NL genes and non-NL genes, respectively (**c**). (**d**) Scatter plot of RNA-seq gene expression against GRO-seq gene activity. NL genes are shown as red dots while all other genes are shown as black dots. Spearman’s correlation coefficients between the two variables are 0.79 for all genes, 0.67 for non-NL genes and 0.56 for NL genes (p-values < 2.2e-16). Blue dashed lines indicate cut-off values for defining active genes and expressed genes, respectively. (**e**) Numbers and percentages of genes grouped by GRO-seq gene activity and RNA-seq expression. (**f**) Box plot comparing gene activities between active NL genes and active non-NL genes.

We measured genome-wide transcriptional activities in C2C12 myoblasts by global run-on sequencing (GRO-seq)^15^, which provided extremely high sensitivity and precision in detecting the locations, densities and orientations of transcriptionally-engaged RNA polymerase II (Pol II). The majority (90.8%) of GRO-seq reads were aligned to the sense strand of annotated RefSeq genes, 3.7% corresponded to post-poly(A) transcription, 0.6% corresponded to divergent transcription upstream of TSS^15^, while the remaining 4.9% of reads were localized to intergenic regions (Fig. 1a,b). GRO-seq read densities of NL genes were at least an order of magnitude lower than those of non-NL genes (Fig. 1c). The transcriptional activity of a gene (referred to as gene activity below) was defined as GRO-seq read density (reads per kilo mappable base per million, RPK_m_M) along the sense strand of the gene body^15^, which was significantly correlated (Spearman’s ρ = 0.79) with gene expression (fragments per kilobase of exon per million, FPKM) measured by RNA-seq^18, 19^ (Fig. 1d). GRO-seq identified 16,957 active genes (70.2%), among which 3,663 genes (21.6%) were considered not expressed by RNA-seq (Fig. 1e). As expected, NL genes have a significantly lower fraction of active genes than non-NL genes (Fig. 1e, Fisher’s exact test, p-value < 2.2e-16). Notably, GRO-seq identified that 745 NL genes (21.9%) were actively transcribed while RNA-seq only identified 336 expressed NL genes (9.9%) (Fig. 1e). Therefore, GRO-seq outperformed RNA-seq in detecting transcription from NL genes that generally have much lower gene activities than non-NL genes (Fig. 1f, Mann-Whitney test, p-value = 8.5e-254).

**Figure 2:**
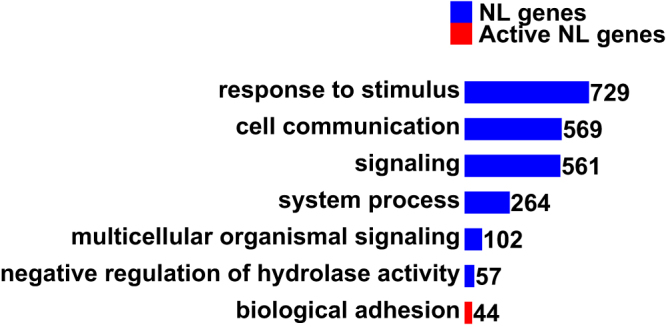
GO analysis of NL genes. The presented Biological Process categories are enriched with Family Discovery Rates (FDRs) < 0.05. Bar lengths are proportional to gene numbers (displayed on the right end). Note that some child GO categories of the displayed parent GO categories are also enriched (see the complete list in Supplementary Table S4).

Gene Ontology (GO) analysis revealed that NL genes are enriched in a list of biological processes (Fig. 2, Supplementary Table S4) including response to stimulus, cell communication, signalling, ion transport, biological adhesion, etc.

### NL-promoter interactions correlate with transcriptional repression of NL genes

**Figure 3:**
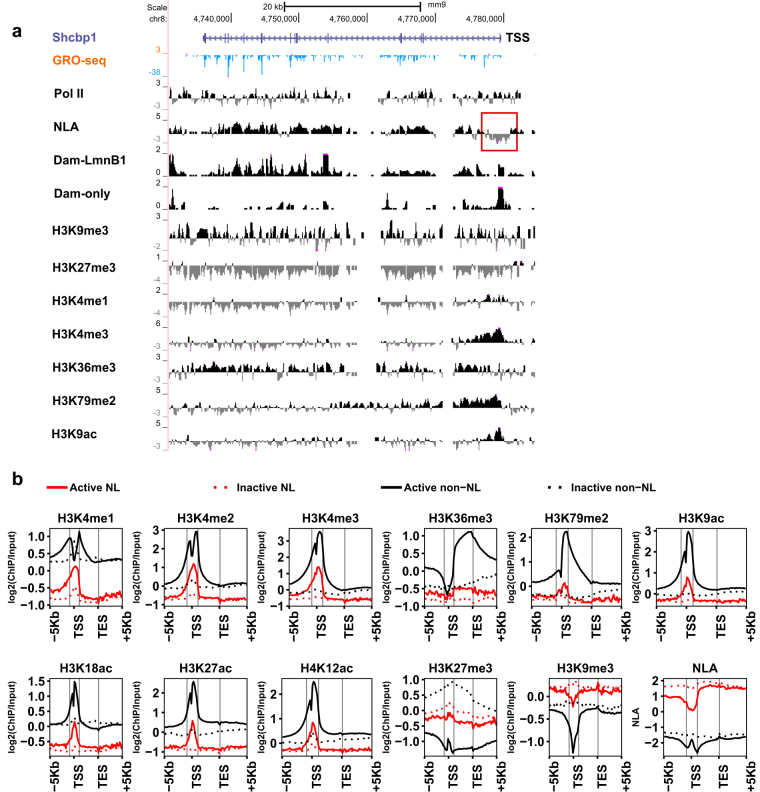
Combined analyses of gene activity, NLA and histone marks across genic regions. (**a**) The UCSC browser view of *Shcbp1* – an active NL gene in C2C12 myoblasts. Data are binned in 200 bp. Tracks “Dam-LmnB1” and “Dam-only” display read densities (RPKM) of Dam-LmnB1 and Dam-only, respectively. Tracks of Pol II and histone marks are log2 of ChIP-Input enrichment computed from ChIP-seq data^21, 22^. The negative NLA values (shown as grey signals in the red square) around TSS in track “NLA” indicate NLA reduction at its promoter. (**b**) Comparing average levels of histone marks and NLA across genic regions (from TSS −5 Kb to TES + 5 Kb) among active/inactive NL and non-NL genes. Data are computed based on 20 equally-divided bins along gene bodies or 200 bp bins otherwise. In each plot, grey vertical lines from left to right indicate TSS −1 Kb, TSS + 1 Kb and TES, respectively.

Next, we analysed DamID-seq data in conjunction with GRO-seq data in order to study the potential roles of NL in regulating native NL gene transcription. Figure 3a and Supplementary Figure S1 display genome browser views of an active NL gene *Shcbp1* in C2C12 myoblast cells and NIH 3T3 fibroblast cells, respectively. Note that the promoter region (TSS ± 1 kb) of *Shcbp1* showed negative NLA values while the gene body region showed positive NLA values in both C2C12 myoblasts and 3T3 fibroblasts (Fig. 3a, Supplementary Fig. S1). Indeed, we found that many other active NL genes showed negative NLA at their promoter regions (additional examples are provided in Supplementary Fig. S2). In C2C12 cells, 345 (46.3%) active NL genes have negative NLA and 246 (9.3%) inactive NL genes have negative NLA (Supplementary Table S5). Fisher’s exact test revealed that active NL genes are more likely to have negative NLA at their promoters than inactive NL genes (p-value = 1.3e-104).

To substantiate our finding, we analysed previously published data on genome-NL association and gene expression. We found that expressed NL genes were more likely (13.7% versus 6.1%) to have promoter regions located outside LADs^5^ than non-expressed NL genes in NIH 3T3 fibroblasts (Supplementary Table S3, p-value = 8.0e-6 by Fisher’s exact test). In addition, we identified a higher fraction of expressed NL gene have negative NLA than non-expressed NL genes from both DamID-seq and from DamID-microarray data^5^ in NIH 3T3 fibroblasts (Supplementary Table S5). Furthermore, we analysed published Lamin B1 ChIP-seq data in mouse ES cells^20^ and detected lower Lamin B1 occupancy at promoters of expressed NL genes than at non-expressed NL genes (data not shown). Taken together, our analysis indicated a unique feature of active/expressed NL genes that their promoter regions are more likely to be excluded from NL association while their gene bodies remain NL-associated.

As another test to examine whether prNLA may regulate gene activity, we analysed 15,712 non-NL genes (among which the effect of gbNLA on transcription should be minimal) including 83 genes with high prNLA (≥1) and 14,393 genes with low prNLA (<−1). The high prNLA group, compared with the low prNLA group, had a higher fraction of inactive genes (38.6% versus 13.2%, p-value = 7e-9 by Fisher’s exact test) and lower gene activities among active genes (median of 0.6 versus 1.1, p-value = 6e-4 by Mann-Whitney test). Thus, increased promoter-NL associations may repress transcription of non-NL genes.

We have considered the potential causes of negative NLA at NL gene promoters. We first examined the contributions of NL contact and chromatin accessibility and made the following observations: 1) Average Dam-LmnB1 read densities both at promoter regions and at upstream regions (up to TSS −5 kb) were lower among active NL genes than inactive NL genes (Supplementary Fig. S3). 2) Dam-only read densities were higher at active NL gene promoters than at inactive NL gene promoters (Supplementary Fig. S3), indicating higher DNA accessibility to free Dam proteins at promoter regions. 3) After normalizing Dam-LmnB1 read densities to those of wild-type C2C12 cell genomic DNA in order to correct for potential sequencing biases, we still observed lower Dam-LmnB1 read densities at promoter and upstream regions of active NL genes (Supplementary Fig. S3). Therefore, the observed negative NLA at promoter regions of active NL genes results from both reduced NL contact and increased DNA accessibility to free Dam proteins.

Because DamID signals depend on available GATC motifs in the genome, we analysed GATC motifs among NL genes and identified two regions slightly deficient in GATC motifs around TSS and TES of both active and inactive NL genes (Supplementary Fig. S3). However, GATC motif deficiency at TSS was in contrast with the good Dam-only signals consistently observed at active NL gene promoters (Fig. 3a, Supplementary Figs S1 and S2). Therefore, it is unlikely that DamID procedures gave rise to the observed negative NLA at active NL gene promoters.

Taken together, negative NLA at active NL gene promoters indicates the following scenarios: 1) Active NL genes are less tightly associated with the NL at their promoters than at gene bodies. 2) Active NL gene promoters remain associated with the NL but are more accessible to Dam-only enzymes due to their open chromatin structures. 3) Active NL gene alleles (in particular promoter regions) may be positioned in proximity to the NL at a lower frequency than inactive NL genes alleles. It would be interesting to test whether active transcription results in reduced NL-promoter interaction or contact frequency, and whether forced or reduced NL-promoter contact can modulate transcriptional output.

### Chromatin features correlate with transcriptional activities among NL genes

To understand how NL-association may affect the epigenetic landscape at genic regions, we analysed the distributions of major histone marks^21–23^ across active and inactive NL/non-NL genes. Levels (ChIP/Input enrichment) of all examined active histone marks are significantly lower among active NL genes than active non-NL genes (Fig. 3b), consistent with the facts that NL genes are transcribed at a lower level (Fig. 1c,f). Surprisingly, although the repressive histone mark H3K27me3 is present in LADs and enriched at LAD borders^4, 14^, H3K27me3 is on average not enriched at NL genes and inactive non-NL genes have higher H3K27me3 levels than inactive NL genes (Fig. 3b), which will be discussed in a later section. H3K9me3, another repressive histone mark associated with LADs, is moderately enriched at both active and inactive NL genes (less than 1.3-fold enrichment across genic regions, Fig. 3b) in contrast to NLA (ranging from 1.9-fold to 3.7-fold enrichment along gene bodies, Fig. 3b). We note that promoter regions of active NL genes have lower H3K9me3 levels than gene bodies (Fig. 3b), which is congruent with the observed NLA reduction at promoters (Fig. 3a,b).

Next, we calculated Spearman’s correlations between each histone mark/NLA and gene activity to find the genic region that each histone mark is most strongly correlated with transcription. In general, regions displaying the strongest positive correlations between gene activity and active histone marks (Supplementary Fig. S3) are congruent with their peak locations (Fig. 3b). Gene activity is anti-correlated with NLA, H3K9me3 and H3K27me3 levels throughout the genic region with distinct correlation patterns (Supplementary Fig. S3). Gene activity has the strongest anti-correlations with H3K27me3 along gene bodies but has the strongest anti-correlations with H3K9me3 at promoter regions (Supplementary Fig. S3). Among active NL genes, the anti-correlation between gene activity and NLA is the strongest at promoter and upstream regions (TSS −5 kb to TSS + 1 kb) and diminishes towards the TES (Supplementary Fig. S3), supporting the notion that NL-promoter interactions may repress transcription.

We obtained consistent results by analysing NLA, histone mark ChIP-seq and RNA-seq data in NIH 3T3 fibroblasts (Supplementary Fig. S1)^14, 24–26^. Taken together, our analyses revealed that NL genes have lower levels of active histone marks than non-NL genes (even in the absence of transcription), moderately enriched with H3K9me3, and on average not enriched with H3K27me3. Our analyses further suggested that H3K27me3, H3K9me3 and NLA may differentially regulate gene repression.

### RNA Pol II promoter-proximal pausing in NL genes

**Figure 4:**
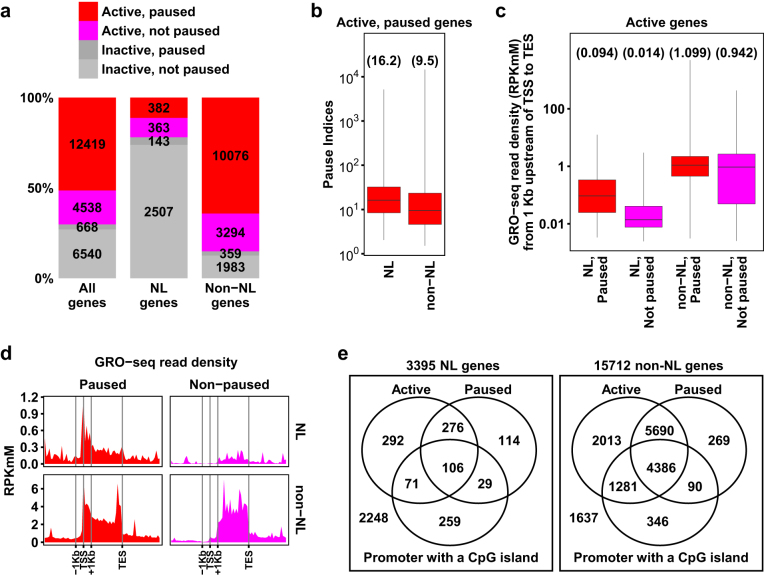
Detecting RNA Pol II promoter-proximal pausing among NL genes. (**a**) Numbers and percentages of genes grouped by gene activity, NL-association and Pol II pausing status. (**b**) Box plot comparing pause indices between active, paused NL genes and active, paused non-NL genes. (**c**) Box plot comparing Pol II recruitment levels among active NL genes and active non-NL genes grouped by their pausing status. In panels b and c, the minimum and the maximum values are presented by whisker ends and the quartiles by box boundaries. The median values are shown in parentheses. Paused genes have significantly higher Pol II recruitment than non-paused genes among both NL and non-NL genes (Mann-Whitney test, p-values < 2.2e-16). (**d**) Average GRO-seq read densities along genic regions of paused and non-paused NL/non-NL genes. Data are binned as described in Fig. 3b. (**e**) Venn diagrams showing distributions of NL and non-NL genes according to gene activity, RNA Pol II pausing and CpG island-containing promoters. Genome coordinates of CpG islands in mouse genome assembly mm9 were downloaded from UCSC genome browser. A CpG island-containing promoter was defined by the existence of a CpG island within ±1 Kb of a TSS^15^.

In addition to quantifying gene activity, GRO-seq allows probing RNA Pol II distributions across genic regions and examining promoter-proximal pausing of RNA Pol II – a major gene regulatory step in metazoans^27^. We found that 12,419 (73.2%) of active genes but only 668 (9.3%) of inactive genes had paused Pol II in C2C12 myoblasts (Fig. 4a), consistent with a previous GRO-seq analysis in mouse cells^16^. Paused Pol II was identified among 382 (51.3%) of active NL genes and 143 (5.4%) of inactive NL genes (Fig. 4a), indicating that Pol II pausing also plays a role in regulating NL gene transcription. We found that active paused NL genes had higher pause indices (PI) than active paused non-NL genes (Fig. 4b; Mann-Whitney test, p-value = 1.8e-19). Given that NL genes have lower transcriptional activities than non-NL genes (Fig. 1c,f), this finding was consistent with the previous study reporting higher PIs among genes with lower activities in human cells^15^. However, there were no significant differences in PI between active paused NL genes and active paused non-NL genes grouped at the same gene activity level (Supplementary Fig. S4). Therefore, PI of active NL genes are likely affected by their lower gene activities but not by NLA.

Next, we compared Pol II recruitment levels (GRO-seq read density from TSS −1 Kb to TES) among paused and non-paused NL/non-NL genes. NL genes had lower Pol II recruitment levels than non-NL genes regardless of pausing status (Fig. 4c,d). Strikingly, the median Pol II recruitment level was ~7-fold higher among active paused NL genes than active non-paused NL genes but was similar between active paused non-NL genes and active non-paused non-NL genes (Fig. 4c,d). Thus, our findings indicate that Pol II promoter-proximal pausing and Pol II recruitment are correlated among active NL genes. Higher Pol II recruitment may result in the formation of paused Pol II at NL genes, while the release of paused Pol II at NL genes (indicated by PI) may be regulated similarly as non-NL genes.

Moreover, CpG islands reside on promoter regions of 465 (13.7%) NL genes and 6,103 (38.8%) non-NL genes (Fig. 4e), indicating that NL genes are less likely to have a promoter CpG island than non-NL genes (Fisher’s exact test, p-value = 1.3e-194). Among non-NL genes with promoter CpG islands, 5,667 (92.9%) are active and 4,476 (73.3%) are paused (Fig. 4e), consistent with the co-occurrence of these transcriptional features in human cells^15^. In contrast, among NL genes with promoter CpG islands, 177 (38.1%) are active and 135 (29.0%) are paused (Fig. 4e), indicating that the co-occurrence of these three transcriptional features among NL genes, while still exists, is less eminent than non-NL genes (Fisher exact test, all p-values < 9.7e-16).

### Five gene classes in the mouse genome exhibit distinct epigenetic features

**Figure 5:**
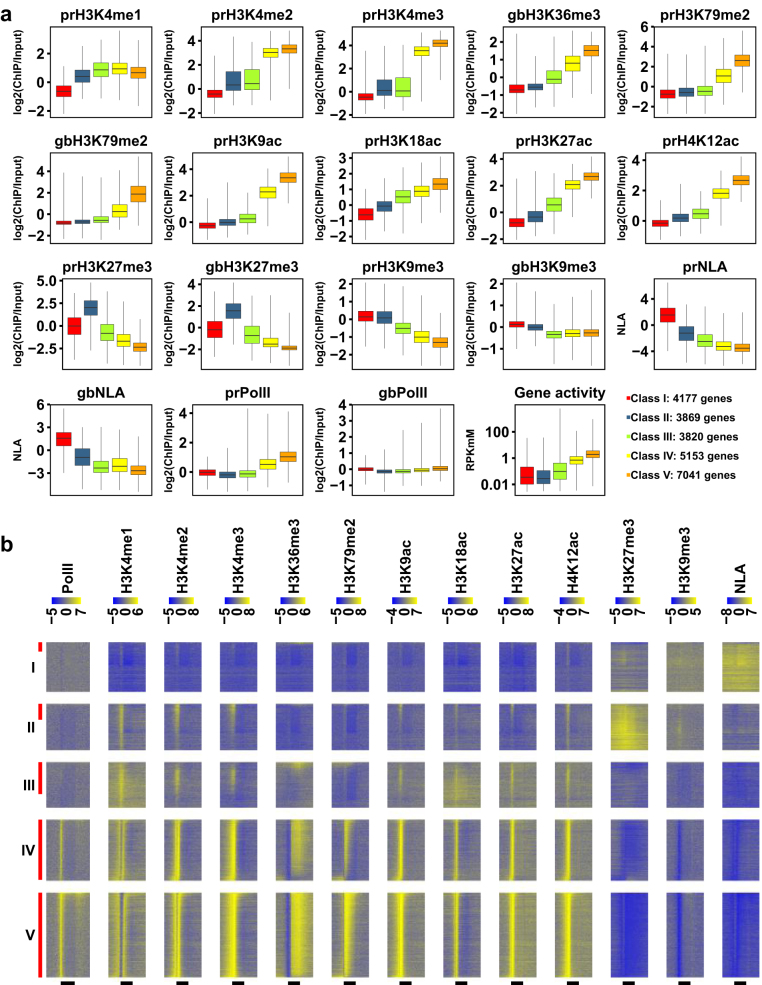
Five gene classes identified by clustering analysis exhibit distinct epigenetic features. (**a**) Box plots comparing levels of histone marks, NLA, and gene activity among the five gene classes in C2C12 myoblasts. “pr” and “gb” represent promoter regions and gene bodies, respectively. The minimum and the maximum values are presented by whisker ends and quartiles by box boundaries. For all variables, Mann-Whitney tests revealed significant differences (FDRs < 0.05) between every pair of the five classes. (**b**) Heat maps of Pol II, histone marks and NLA across genic regions. Genes in each class are sorted by decreasing gene activity. Vertical red bars on the left mark active genes (18.58%, 33.95%, 69.88%, 98.50% and 99.97% of Class I to V, respectively). Horizontal black bars at the bottom mark annotate transcribed regions from TSS to TES. Data are binned as described in Fig. 3b.

To gain a genome-wide view on how epigenetic features including NLA regulate gene expression, we performed principal component analysis (PCA) and k-means clustering on histone modification ChIP-seq data^21–23^ and NLA DamID-seq data^14^ at genic regions in C2C12 myoblasts. We clustered 24,165 mouse RefSeq genes into five distinct classes (Fig. 5). Class I and Class II genes have the highest levels of NLA and H3K27me3, respectively (Fig. 5a). Both gene classes have low gene activities and low levels of active histone marks (Fig. 5a, Supplementary Table S6). These results indicate that at the genome-wide level, NLA and H3K27me3 represent two major types of repressive chromatin at genic regions. This finding is generally congruent with the previous finding of several distinct repressive chromatin types in *Drosophila* cells^28, 29^. Nonetheless, because Class I genes on average are moderately enriched with H3K9me3 (Fig. 5a,b), a major portion of Class I genes may exhibit heterochromatin features and differ from the BLACK chromatin identified in *Drosophila* ^28^. Importantly, we identified a similar set of five gene classes using ChIP-seq and DamID-seq data in NIH 3T3 fibroblasts^14, 24, 25^ (Supplementary Fig. S5, Supplementary Table S6), indicating the robustness of our clustering analysis and thus confirming our findings.

GO analysis revealed that Class I and II genes are enriched in distinct biological processes. Class I genes are uniquely enriched in sensory perception, defence response to bacterium and biological adhesion (Supplementary Fig. S6, Supplemental Table S4), which is consistent with our GO analysis of NL genes (Fig. 2, Supplementary Table S4). Class II genes are uniquely enriched in developmental processes (e.g. cell differentiation and nervous system development) and behaviour (Supplementary Fig. S6, Supplementary Table S4).

### NL-association represses active histone marks and gene activity

**Figure 6:**
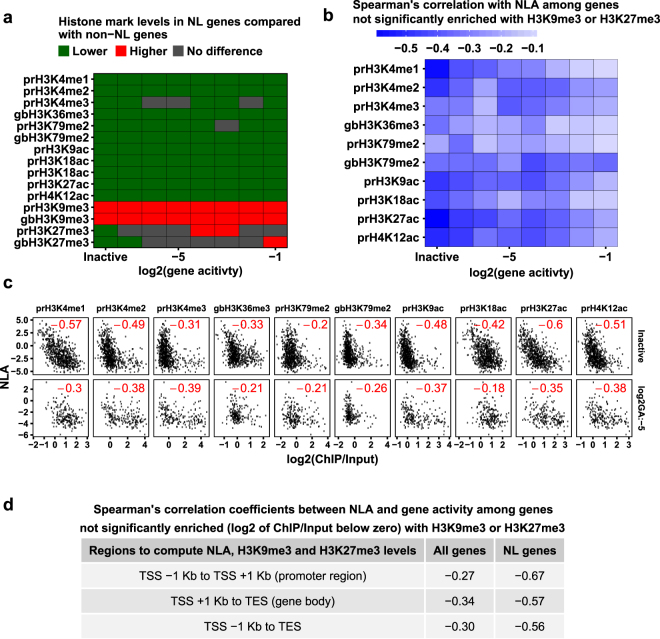
Anti-correlations between NLA and active histone marks or gene activity. (**a**) The heat map displaying comparisons of histone mark levels between NL and non-NL genes grouped by gene activity. Colours indicate that histone mark levels are higher (red), lower (green) or not significantly different (grey) among NL genes compared to non-NL genes as revealed by Mann-Whitney test (cut-off FDR at 0.05). Gene activity groups are defined by rounding log2 of gene activity (RPK_m_M) to the nearest integer. Each analysed group has 53 NL genes and 451 non-NL genes at minimum. (**b**) The heat map displaying Spearman’s correlation coefficients between NLA and each active histone mark among genes that are not significantly enriched (log2 of ChIP-Input ratio below zero) with H3K9me3 or H3K27me3 across genic regions. FDRs < 0.05 in all cases. (**c**) Scatter plots of NLA against each active histone mark at selected gene activity levels among genes that are not significantly enriched with H3K9me3 or H3K27me3. Spearman’s correlation coefficients are shown in red. In panels b and c, gene activity groups are defined as in panel a. The analysed groups have 173 genes at minimum. (**d**) A table showing negative correlations between NLA and gene activity among all genes or NL genes not significantly enriched with H3K9me3 or H3K27me3.

Because NL genes display much lower levels of active histone marks (Fig. 3b) and lower gene activities than non-NL genes (Fig. 1f), we sought to determine whether lower gene activities can fully explain the reduced levels of active histone marks at NL genes. If so, we would expect that NL and non-NL genes transcribed at the same level should accommodate similar levels of active histone marks. However, Mann-Whitney tests revealed that all active histone mark levels were significantly lower in NL genes than non-NL genes at the same gene activity level (Fig. 6a). Therefore, active histone marks are repressed at both active and inactive NL genes. For repressive marks, H3K9me3 levels were always higher in NL genes than non-NL genes (Fig. 6a). In contrast, H3K27me3 levels were lower in inactive NL genes than inactive non-NL genes and were mostly not different between active NL and active non-NL genes (Fig. 6a).

Next, we calculated Spearman’s correlations among genes not significantly enriched with H3K9me3 or H3K27me3 (log2 of ChIP/Input below zero) and found negative correlations between each active histone mark and NLA at all gene activity levels (Fig. 6b,c). Similarly, we found negative correlations between gene activity and NLA (in promoter regions, gene bodies or combined regions) among genes not significantly enriched with H3K9me3 and/or H3K27me3 (Fig. 6d). In particular, promoter NL-association has a much stronger negative correlation with gene activity among NL genes (Spearman’s ρ = −0.67) than among all genes (Spearman’s ρ = −0.27), further supporting its function in repressing NL gene transcription. Therefore, our analysis has revealed that NL-association represses both active histone marks and transcriptional activities.

### NL gene subgroups are differentially enriched with heterochromatic histone marks

**Figure 7:**
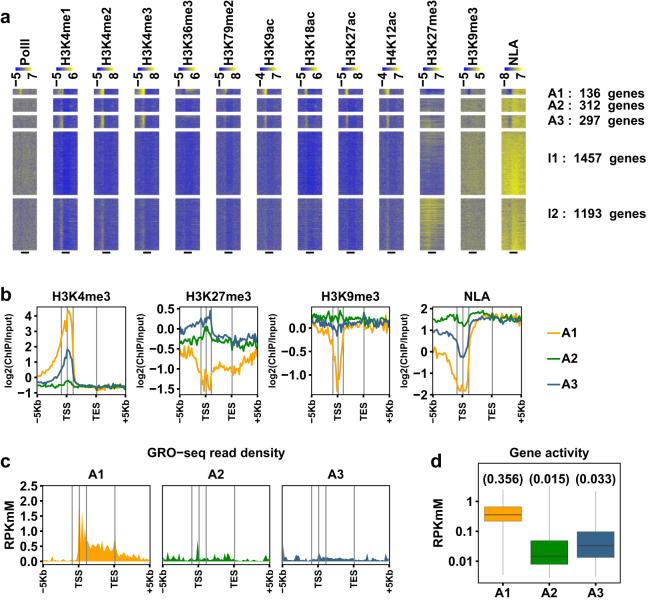
NL genes are differentially enriched with heterochromatic histone marks. (**a**) Heat maps displaying levels of Pol II, histone marks and NLA across genic regions among three groups (A1-A3) of active NL genes and two groups (I1-I2) of inactive NL genes. Genes in A1-A3 groups are sorted by decreasing gene activity and in I1-I2 by increasing gene body NLA. Black bars at the bottom mark annotated transcribed regions from TSS to TES. (**b**,**c**) Plotting average levels of selected histone marks and NLA (**b**) or average GRO-seq read density (**c**) along genic regions of A1-A3 gene groups, respectively. In a-c, data are binned as described in Fig. 3b. (**d**) Box plot comparing gene activities among A1-A3 gene groups. The minimum and the maximum values are presented by whisker ends and the quartiles by box boundaries. The median values are shown in parentheses.

Several studies have reported that Lamin B1-interacting genomic regions largely overlap with heterochromatin^4, 8, 30^. To examine differential distributions of active and repressive histone modifications among NL genes, we clustered active NL genes into three groups (A1-A3) and inactive NL genes into two groups (I1-I2), respectively (Fig. 7a, Supplementary Fig. S7, Supplementary Tables S1 and S7). A1 group (18.3% of active NL genes) has the highest gene activities (Fig. 7c,d) and the highest levels of active histone marks at promoter regions, and in general is not enriched with H3K27me3 or H3K9me3 (Fig. 7b, Supplementary Fig. S7, Supplementary Table S7). Genome browser views of NLA and epigenetic features of three A1 genes (*Shcbp1*, *Caprin2* and *Sp4*) are provided in Fig. 3a and Supplementary Fig. S2. A2 and A3 genes are similar in group sizes (41.9% and 39.9% of active NL genes, respectively), display gene activities that are at least one order of magnitude lower than A1 (Fig. 7c,d) and are slightly enriched (less than 1.2-fold) with H3K9me3 across genic regions (Fig. 7b, Supplementary Fig. S7, Supplementary Table S7). Interestingly, A3 genes have higher levels of both H3K27me3 and active histone marks in promoter regions as well as higher gene activities than A2 genes (Fig. 7b,d, Supplementary Fig. S7, Supplementary Table S7). A1, A2 and A3 groups also differ in the fraction of paused genes (87.2%, 36.5% and 51.3% respectively; p-values < 0.0002 from pairwise comparison by Fisher’s exact test) and PIs among paused genes (median of 14.4, 22.4 and 16.1, respectively; p-values < 0.01 from pairwise comparison by Mann-Whitney test). Among inactive NL genes, both I1 and I2 genes are enriched with H3K9me3 (slightly higher in I1) and I2 genes are more strongly enriched with H3K27me3 (Fig. 7a, Supplementary Figs S7 and S8, Supplementary Table S7). Genome browser views of NLA and epigenetic features of one A2 gene (*Trdn*), one A3 gene (*Pawr*), one I1 gene (*Foxp2*) and one I2 gene (*Tbx5*) are provided in Supplementary Fig. S8. Therefore, a subset of both active NL genes and inactive NL genes are enriched with H3K27me3. Note that these findings reconcile with previous results that H3K27me3 are present in LADs and are enriched on LAD borders^4, 30^.

We repeated the above analyses in NIH 3T3 fibroblasts and identified similar NL gene subgroups where groups E1-E3 of expressed NL genes resembled A1-A3 in C2C12 myoblasts and groups N1-N2 of non-expressed NL genes resembled I1-I2 in C2C12 myoblasts (Supplementary Fig. S9, Supplementary Table S7). Further analyses revealed that E1 genes are more likely to have their TSSs located outside of LADs (Supplementary Fig. S9). In summary, for the first time, we identified NL gene subgroups that are differentially enriched with heterochromatic histone marks. A significant fraction of active NL genes (A1) is not enriched with either H3K9me3 or H3K27me3 and transcribed at relatively high levels. Moreover, one active NL gene subgroup (A3) and one inactive NL gene subgroup (I2) are enriched with H3K27me3, suggesting that H3K27me3 may regulate these NL genes.

**Figure 8:**
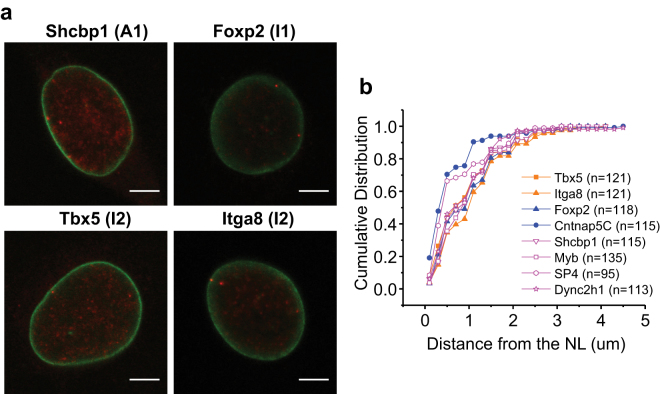
NL genes of different subgroups are localized to the nuclear periphery. (**a**) Representative DNA FISH images of one A1 gene (*Shcbp1*), one I1 gene (*Foxp2*) and two I2 genes (*Tbx5* and *Itga8*). FISH signals are shown in red and anti-Lamin B immunofluorescence signals were shown in green. Scale bars: 5 μm. (**b**) Cumulative frequency distributions of distances between eight NL genes and the NL. A1, I1 and I2 genes were plotted in pink, blue and orange, respectively. Pair-wise Kolmogorov-Smirnov (K-S) tests among six NL genes (*Shcbp1*, *Myb*, *Dync2h1*, *Foxp2*, *Tbx5* and *Itga8*): P > 0.05 in all cases. Pair-wise K-S test between *Sp4* or *Cntnap5C* and each of the other six NL genes: P < 0.05 in all cases. FISH data of three A1 genes (*Myb*, *Sp4* and *Dync2h1*) and one I1 gene (*Cntnap5C*) were obtained from our previous study^14^.

We next examined the subnuclear localizations of several NL genes of different subgroups by fluorescence *in situ* hybridization (FISH). All eight examined NL genes are preferentially localized to the nuclear periphery (Fig. 8a)^14^. Interestingly, two I2 genes (*Tbx5* and *Itga8*), one I1 gene (*Foxp2*) and three A1 genes (*Shcbp1*, *Myb* and *Dync2h1*) show indistinguishable cumulative frequency distributions from the NL (Fig. 8b), suggesting that absolute distances from the NL do not determine transcriptional status or epigenetic modifications of NL genes. The remaining two genes, *Cntnap5C* (I1) and *Sp4* (A1), show higher frequency of alleles localized to the NL (Fig. 8b), although their gbNLA values are not higher than those of the above six genes. Thus, genes within each NL gene subgroup may slightly differ in their subnuclear localizations, but this does not appear to correlate with gene activity or NL-association quantified by DamID-seq.

### Putative enhancers for NL genes and their epigenetic features

To explore whether active NL genes may be regulated by distal enhancers, we analysed two sets of enhancers in C2C12 myoblasts – 4,315 enhancers detected based on epigenomic data^23^ which will be referred to as Blum enhancers, and 5,570 putative enhancer sites identified using our GRO-seq data and the dREG method developed by Danko *et al.* ^31^, which will be referred to as dREG enhancers (Supplementary Table S8). The two methods apparently identified two distinct sets of enhancers in that only 182 pairs of Blum enhancer or dREG enhancer for the same gene are located within 5 Kb distance. For the subsequent analyses, we first excluded enhancers that were located within 1 Kb of a TSS and associated each gene with its nearest enhancer if the TSS-enhancer distance was shorter than 200 Kb. As a result, 10,715 genes were associated with 2,671 Blum enhancers and 10,336 genes were associated with 1,983 dREG enhancers, where 5,455 genes were in common and hence had both types of enhancers. Particularly, 99 active NL genes were associated with 74 dREG enhancers and 112 active NL genes were associated with 93 Blum enhancers (Supplementary Table S9). Note that multiple genes may be associated with the same enhancer. The following analyses will focus on these two subsets of enhancers and their potential target genes (Supplementary Table S9).

We first examined whether the presence of a nearby enhancer is linked to higher gene activity. For both active NL genes and active non-NL genes, existence of dREG enhancers but not Blum enhancers, was associated with higher gene activities (Supplementary Figs S10 and S11). Next, we examined whether the distance between enhancers and TSS is linked to gene activity. Interestingly, TSSs of active genes tend to be located closer to their enhancers (both Blum and dREG enhancers) than TSSs of inactive genes regardless of NL association (Supplementary Figs S10 and S11). Moreover, distances between TSSs and enhancers of active NL genes (with lower gene activities, Fig. 1f) are longer than distances between TSSs and enhancers of active non-NL genes (Supplementary Figs S10 and S11). Therefore, a shorter distance between enhancer and TSS of a gene appears to be correlated with higher gene activity.

Next, we examined the epigenetic landscapes of enhancers associated with active NL genes and active non-NL genes. Both dREG enhancers and Blum enhancers display active histone marks peaked at enhancer centre (Supplementary Figs S10 and S11). Interestingly, dREG enhancers display wider peaks of active histone marks (such as H3K4me1, H3K4me2, H3K27ac) than Blum enhancers (Supplementary Figs S10 and S11). As expected, there are no enrichment of H3K27me3, H3K9me3 or NLA at both enhancer sets (Supplementary Figs S10 and S11). We found that GRO-seq read densities were one order of magnitude higher along dREG enhancers than Blum enhancers (Supplementary Figs S10 and S11), consistent with the facts that dREG enhancers were detected by GRO-seq data^31^ and the two enhancer sets are distinct in their genomic locations. Likewise, H3K36me3 and H3K79me2, two transcription-associated active histone marks, were enriched in dREG enhancers but not in Blum enhancers (Supplementary Figs S10 and S11). Interestingly, dREG enhancers associated with active NL genes display lower peak levels of active histone marks and higher NLA than dREG enhancers associated with active non-NL genes (Supplementary Fig. S10). In contrast, peak levels of Blum enhancers associated with active NL genes are comparable to those Blum enhancers associated with active non-NL genes (although the baseline level – 3 kb away from the enhancer centre are still lower for Blum enhancers associated with active NL genes, Supplementary Fig. S11). Thus, enhancer transcription is a more prominent signature of dREG enhancers and may be more critical for their functions, while active histone marks are more prominent signatures of Blum enhancers and may be more critical for their functions. Further studies are needed to determine the functions of dREG enhancers and Blum enhancers in regulating active NL genes.

Although the majority of dREG enhancers or Blum enhancers lacks NL-association, we noticed that a small number (less than 1% in both sets) of enhancers have NLA higher than 1 and defined those as NL enhancers (Supplementary Figs S10 and S11). We found that NL dREG enhancers had comparable GRO-seq read densities as non-NL dREG enhancers (Supplementary Fig. S10). Interestingly, NL dREG enhancers were depleted of most active histone marks except H3K36me3 (Supplementary Fig. S10), again suggesting that NL dREG enhancers may function through enhancer transcription. In contrast, NL Blum enhancers display peaks of H3K4me1, H3K18ac and H3K27ac around the enhancer centre (Supplementary Fig. S11). Both NL dREG enhancers and NL Blum enhancers show slight enrichment of H3K9me3 but are not enriched with H3K27me3 (Supplementary Figs S10 and S11). Thus, NL enhancers may represent a novel class of enhancers by their unique epigenetic signatures and their functions call for future studies.

### Putative super enhancers (SEs) for NL genes

To obtain a more comprehensive profile of putative enhancer elements, we identified two sets of putative SEs in C2C12 myoblasts using publicly-available MyoD1 and H3K27ac ChIP-seq data^23^ and the ROSE program^32, 33^ and will refer to them as MyoD1 SEs and H3K27ac SEs, respectively (Supplementary Table S10). The 934 MyoD1 SEs ranged from 0.4 to 60.5 Kb with the median at 6.9 Kb, while the 1,050 H3K27ac SEs ranged from 0.7 to 411.7 Kb with the median at 57.1 Kb. The two SE sets shared 361 regions, i.e. two regions overlapped or had boundary-to-boundary distance within 5 Kb. We next associated each gene with its nearest SE if the TSS and the SE boundary are within 200 Kb distance. Thus, 5,129 genes were associated with 840 MyoD1 SEs, 7,523 genes were associated with 1,026 H3K27ac SEs, and 3,275 genes had both types of SEs. Particularly, 31 active NL genes were associated with 27 MyoD1 SEs and 12 active NL genes were associated with 9 H3K27ac SEs (Supplementary Table S11). Further analysis on these two subsets of gene-associated SEs (Supplementary Table S11) revealed that among NL genes, the presence of nearby SEs was not associated with higher gene activities but active genes resided closer to their potential SEs than inactive genes (Supplementary Fig. S12). Like dREG enhancers and Blum enhancers, MyoD1 SEs and H3K27ac SEs were located more distantly from their associated active NL genes than from their associated active non-NL genes (Supplementary Fig. S12). Additionally, we identified two MyoD1 SEs that display positive NLA and H3K4me1 enrichment, but they are located more than 200 kb away from the nearest TSS.

## Discussion

This work has not only confirmed that the NL is permissive for transcription, but also revealed several novel transcriptional features of NL genes in mammalian cells. First, we detected transcriptionally-engaged RNA Pol II in gene bodies of active NL genes, indicating that active NL genes are permissive to transcriptional elongation in the gene body. Second, among active NL genes, NL-associations were often excluded from promoter regions, suggesting that NL-promoter interactions may repress transcription. Third, NLA was anti-correlated with transcription activities among genes not significantly enriched with H3K9me3 or H3K27me3. Therefore, NL-mediated gene repression may occur through a mechanism distinct from heterochromatic histone marks. Fourth, we found that active paused NL genes display much higher Pol II recruitment levels than active non-paused NL genes, suggesting that Pol II recruitment and Pol II promoter-proximal pausing are strongly correlated among active NL genes. In-depth studies will likely reveal functional impacts of the nuclear architecture (i.e., the NL) on specific steps of gene transcription.

Our finding of negative correlations between NL-promoter interaction and gene activity is consistent with a previous report that Lamin A-promoter interaction was anti-correlated with gene expression^34^. Gene repression by NL-promoter interaction may result from regulatory factors residing beneath the inner nuclear membrane (INM). For example, Lamin A and the INM protein Emerin display largely identical genome interactions as Lamin B1^4, 6^ and interact with several transcription repressors^35–38^. Reduced chromatin accessibility^39^ and subnuclear compartmentalization of core promoter factors^40, 41^ may also influence gene transcription. Interestingly, we identified ~100 active NL genes which lack both H3K9me3 and H3K27me3 and have more than 10-fold higher gene activities than the rest of active NL genes (Fig. 7, Supplementary Figs S7 and S9). These genes display high NLA in the gene body but negative NLA at promoters, and will serve as important models to study NL-mediated gene regulation.

Our study suggested potential functions of heterochromatin in regulating NL gene expression. First, NL genes enriched with heterochromatic histone marks H3K9me3 and H3K27me3 have much lower gene activities than NL genes not enriched with these marks (Fig. 7), indicating that these heterochromatic histone marks may confer additional repressive functions to NL genes. Second, H3K27me3 is enriched in ~300 active NL genes (A3) and ~1,200 inactive NL genes (I2) (Fig. 7) and may thus regulate a significant portion of NL genes. Third, NL genes enriched with H3K27me3 also display active histone marks such as H3K4me3 at promoters (Fig. 7b, Supplementary Figs S7 and S8), reminiscent of “bivalent domains” observed in ES cells^42^. Future studies are needed to investigate the roles of NLA, H3K27me3 and H3K9me3 in regulating NL genes and to reveal potential interplays between different gene repression pathways.

Our analysis revealed that NLA was anti-correlated with active histone marks among genes transcribed at the same level and among genes not significantly enriched with H3K9me3 or H3K27me3. Thus, the NL may repress active histone marks independent of transcription and independent of heterochromatin. It will be important to pinpoint the molecular mechanisms repressing active histone marks at the nuclear periphery in mammalian cells. For example, histone modifying enzymes may act at the nuclear periphery to down-regulate active histone marks. Because heterochromatin enrichment facilitates gene positioning to the NL^30, 43–45^, it is also interesting to determine whether disrupting heterochromatin tethering to the NL can alter the polarized distribution of active histone marks.

Finally, we studied regulation of active NL genes by putative enhancers and SEs. We found that distances between enhancers (or SEs) and TSSs were correlated with gene activities. We next examined the epigenetic landscape of enhancers associated with NL genes. Interestingly, dREG enhancers associated with NL genes display enhancer transcription but diminished levels of classical enhancer histone marks (such as H3K4me1 and H3K27ac), and Blum enhancers associated with NL genes are enriched with these active histone marks but display little transcriptional activities. Thus, we identified two classes of distal enhancers that may regulate NL gene transcription through distinct pathways. Furthermore, a small portion of both dREG enhancers and Blum enhancers have high NLA and H3K9me3 enrichment, indicating that NL-associated and H3K9me3-enriched genomic regions are not precluded from acting as distal enhancers.

## Methods

### Selecting genes

Mouse RefSeq genes^17^ on mouse genome assembly mm9 were downloaded from UCSC genome browser database^46^ (http://genome.ucsc.edu). A total of 24,165 genes that meet the following criteria were analysed: genes are located on assembled chromosomes 1 to 19 or X; genes are 2 Kb or longer from TSS to TES; both promoter region and gene body have mappable bases when mapping 75 bp reads; only one copy of the annotated transcripts with the same TSS and TES were retained for analyses.

### GRO-seq assay in C2C12 myoblasts

GRO-seq experiments, data processing, computation of gene activities and detection of active genes and promoter-proximal pausing of RNA Pol II largely followed the published protocols^15, 16^. C2C12 myoblasts cultured to 80% confluency were washed directly on 10 cm plates three times with ice cold 1X PBS. Four 10 cm plates were used for each nuclei isolation. Nuclei were isolated as previously described^15^. GRO-seq libraries were prepared as described^15^ with the following modifications: Total RNA in the 200 μL run-on reaction was extracted by 600 μL Trizol LS (Invitrogen) and subsequently extracted by 160 μL chloroform once, acid phenol-chloroform twice (equal volume) and chloroform once (equal volume). During immunoprecipitation of BrU RNA, 1 μL SUPERaseIN (Ambion, 20 u/μL) was added to 5 mL Binding Buffer. RNA end repair was performed as described in the extraction protocol of NCBI’s Gene Expression Omnibus (GEO)^47^ accession number GSE60454^48^.

Two replicate GRO-seq libraries were generated and sequenced by Illumina’s HiSeq 2000 system. Short reads of 76 bp were mapped onto the mouse genome assembly mm9 by Bowtie2^49^ with the default setting. Uniquely mapped reads with mapping quality ≥40 were used for subsequent analyses. To maximize the yield, unqualified reads in the initial alignment were subject to trimming of 5 bp from the 3′ end and realignment at the reduced length. This process was repeated until reads were trimmed down to 21 bp. Reads mapped to rRNA and tRNA repeats (track RepeatMasker^50^ in UCSC genome browser^46^, http://genome.ucsc.edu) were discarded. The two replicates were pooled for subsequent analyses (Spearman’s ρ = 0.75 based on reads counts in 1 kb non-overlapping contiguous windows along the genome). GRO-seq sequence data can be accessed in GEO through accession number GSE76624.

### Determining gene activities and RNA Pol II pausing by GRO-seq

Gene activity was measured by GRO-seq read density (RPK_m_M) in the sense strand of the gene body^15^. Mappable bases were obtained from the published data^51^. The statistical significance was assessed by comparing to the background with Poisson statistics, where the background was the average read density of 65 gene deserts (1 Mb genomic regions without any UCSC known genes^46, 52^ or rRNA/tRNA repeats^46, 50^). Genes were considered as active with FDRs < 0.01.

In order to examine promoter-proximal pausing of RNA Pol II, the promoter-proximal peak of a gene was identified as the 50 bp window with the highest GRO-seq read density (RPK_m_M) in the sense strand among all 50 bp windows (shifting by 5 bp) of the 2 kb promoter region^15^. Next, Fisher’s exact test was used to compare read counts of the promoter-proximal peak and the gene body against the null hypothesis of a uniform distribution of reads according to mappable bases in the two windows^15^. Genes were considered as paused with FDRs < 0.01.

### Analysing published ChIP-seq and RNA-seq data

Sequence data were downloaded from GEO database and UCSC table browser^53^ (Supplementary Table S12). For ChIP-seq, normalized read densities (RPKM) were used to compute log2 of ChIP-Input enrichment for a genomic region. RNA-seq reads were aligned to the mouse genome assembly mm9 by TopHat^54^. Gene expression levels (FPKM) were computed by Cufflinks^55^. Genes were considered expressed by zFPKM > −3^56^.

### Sequencing genomic DNA of wild-type C2C12 myoblasts and data analysis

A sequencing library was prepared from genomic DNA of wild type C2C12 cells and sequenced using Illumina’s HiSeq 2000 system. All 76 bp single-end reads were mapped onto the mouse genome assembly mm9 using the default setting of Bowtie2^49^. Uniquely mapped reads (one copy is kept at each aligned position) with mapping quality ≥40 were used for analyses. Sequence data can be accessed in GEO through accession number GSE41583.

### Principle Component Analysis (PCA) and k-means clustering

Analyses were performed using R^57^. For C2C12 myoblasts, NLA and log2 of ChIP-Input enrichment of histone marks at promoter regions and/or gene bodies (Fig. 5a) were organized as a matrix of genes in rows and variables in columns and subjected to PCA using R function prcomp (scale = T). The projected data matrix on the first three principal components corresponding to over 80% of variance were then analysed by k-means clustering using R function kmeans (iter.max = 100, nstart = 20, algorithm = “MacQueen”). For clustering of active and inactive NL genes (Fig. 7 and Supplementary Fig. S7), histone mark and NLA data were binned in four windows – 1 Kb region upstream of TSS, 1 Kb region downstream of TSS, the gene body and 1 Kb region downstream of TES. The first 17 (for active NL genes) and the first 23 (for inactive NL genes) principal components corresponding to over 80% of variance were used for k-means clustering. The same analyses were performed for NIH 3T3 fibroblast data (Supplementary Figs S5 and S9). The first four (for all genes), the first 14 (for expressed NL genes) and the first 19 (for non-expressed NL genes) principal components corresponding to over 80% of variance were used for k-means clustering. Each clustering analysis was run with various cluster numbers and the final numbers of clusters reported in this study were determined by easiness of interpretation.

### Analyses of putative enhancers and SEs in C2C12 myoblasts

dREG enhancers were identified using our GRO-seq data in C2C12 myoblasts and the dREG program with its pre-trained model for mammals^31^. dREG peaks ranged from 102 to 3,353 bp. To compare with published Blum enhancers^23^ that were standardized to 5 Kb in length, each dREG peak was extended from its centre to both sides by 2.5 Kb. The centre of an enhancer was used to compute its distance to a TSS. SEs were identified using MyoD1 and H3K27ac ChIP-seq data in C2C12 myoblasts (Supplementary Table S12) and the ROSE program with default settings^32, 33^.

### GO analysis

GO analysis was performed using WEB-based GEne SeT AnaLysis Toolkit^58, 59^. Significance Level was set to 0.05 and Minimum Number of Genes for a Category was set to 10.

### FISH

DNA FISH was performed as described previously^14^. The following bacterial artificial chromosome clones were used to prepare FISH probes: RP23-267A22 (*Shcbp1*), RP23-214K24 (*Foxp2*), RP23-202C2 (*Itga8*), RP23-84F12 (*Tbx5*). A goat-anti-Lamin B antibody (Santa Cruz, sc-6217) was used to mark the nuclear envelope.

## Electronic supplementary material

Supplementary Information

Supplementary Tables S1 to S12
