# Supplementary material for: Identifying Novel Transcriptional and Epigenetic Features of Nuclear Lamina-associated Genes
Source: Sci Rep. 2017 Mar 7;7:100. doi: 10.1038/s41598-017-00176-x (PMC5427898; doi:10.1038/s41598-017-00176-x)
Supplement: Supplementary file 1 — Supplementary Information [file 41598_2017_176_MOESM1_ESM.pdf]

Supplementary Information (SI)

Identifying Novel Transcriptional and Epigenetic Features of Nuclear Lamina-associated Genes

Feinan Wu[1] and Jie Yao[1, *]

[1] Department of Cell Biology, Yale University School of Medicine, New Haven, CT, 06520, USA

* Correspondence should be addressed to J. Y. (Tel: 1-203-737-6897; Fax: 1-203-785-7446;

Email: yao.j@yale.edu).

Supplementary Figures

Figure S1 (NIH 3T3 fibroblasts)

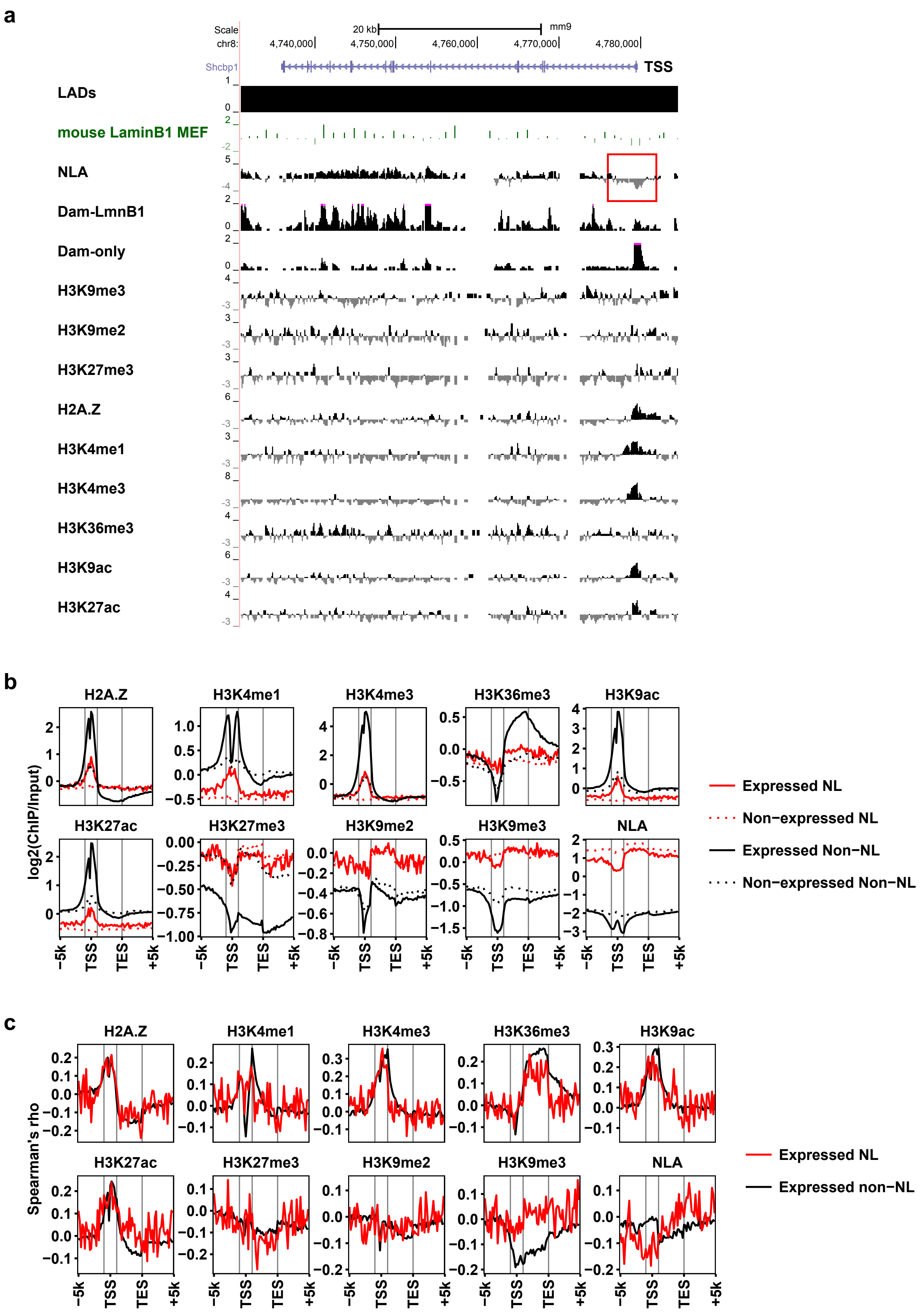

Figure S1. Combined analyses of gene expression, NLA and histone modifications across genic regions in NIH 3T3 fibroblasts. (a) A UCSC genome browser view of *Shcbp1* – an expressed NL gene in NIH 3T3 fibroblasts (MEF). Track “mouse LaminB1 MEF” is publicly available on UCSC genome browser, which shows log2 ratios of Dam-LmnB1 over Dam-only from the previously reported DamID-microarray data in 3T3 cells [1]. *Shcbp1* is located within a LAD as indicated by the black box in track “LADs”. Legends of other tracks follow Fig. 3a. **(b)** Comparing average levels of histone marks and NLA across genic regions (from TSS -5 Kb to TES +5 Kb) among expressed/non-expressed NL and non-NL genes. **(c)** Comparing patterns of Spearman’s correlations of RNA-seq gene expression relative to histone marks and NLA across genic regions between expressed NL genes and expressed non-NL genes. In panels b and c, data are binned as described in Fig. 3b. Grey vertical lines from left to right indicate TSS -1 Kb, TSS +1 Kb and TES, respectively.

Figure S2 (C2C12 myoblasts)

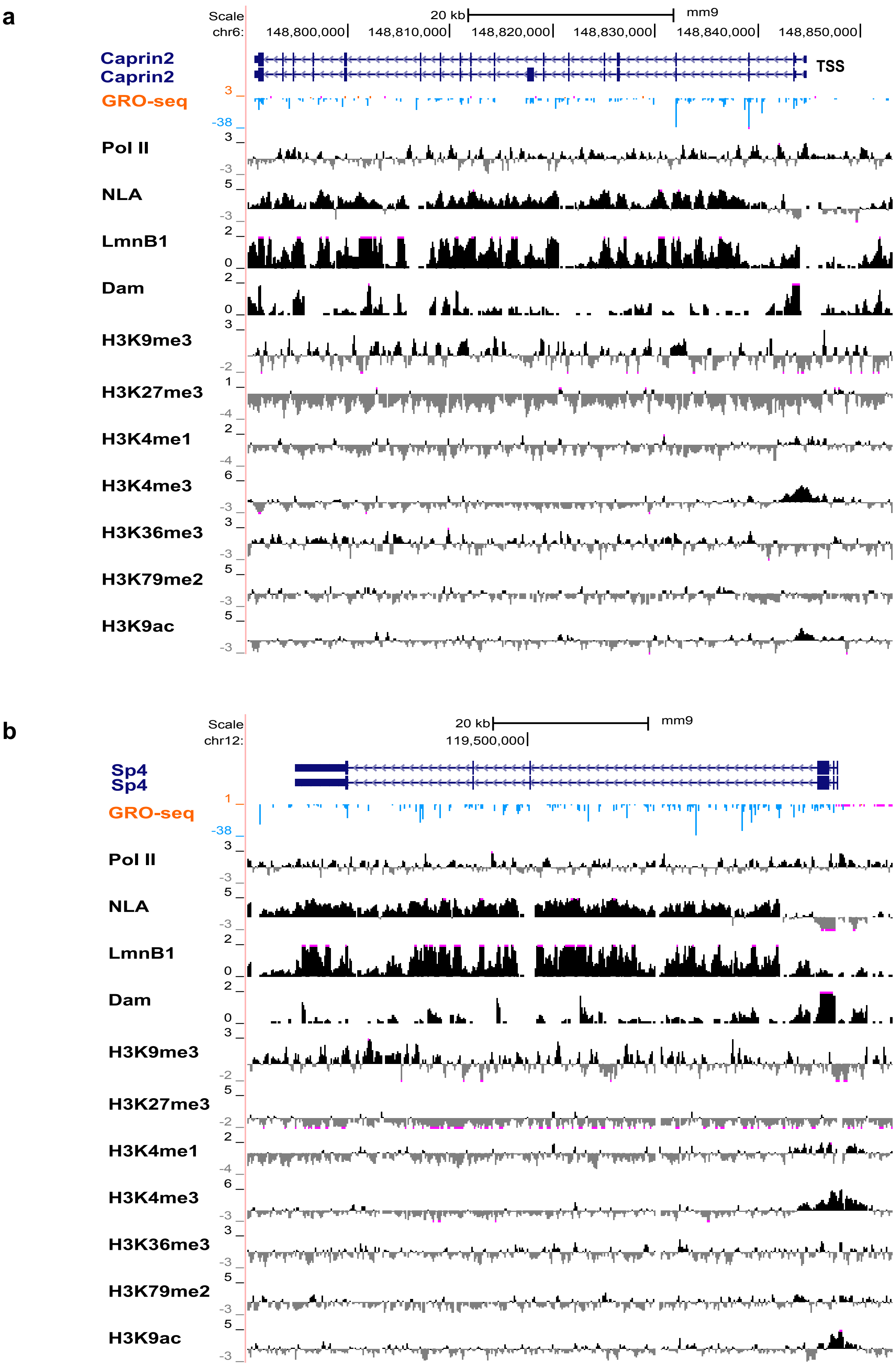

Figure S2. UCSC genome browser views of *Caprin2* (A) and *Sp4* (B) – two active NL genes (A1 group) in C2C12 myoblasts. Legends follow Fig. 3a.

Figure S3 (C2C12 myoblasts)

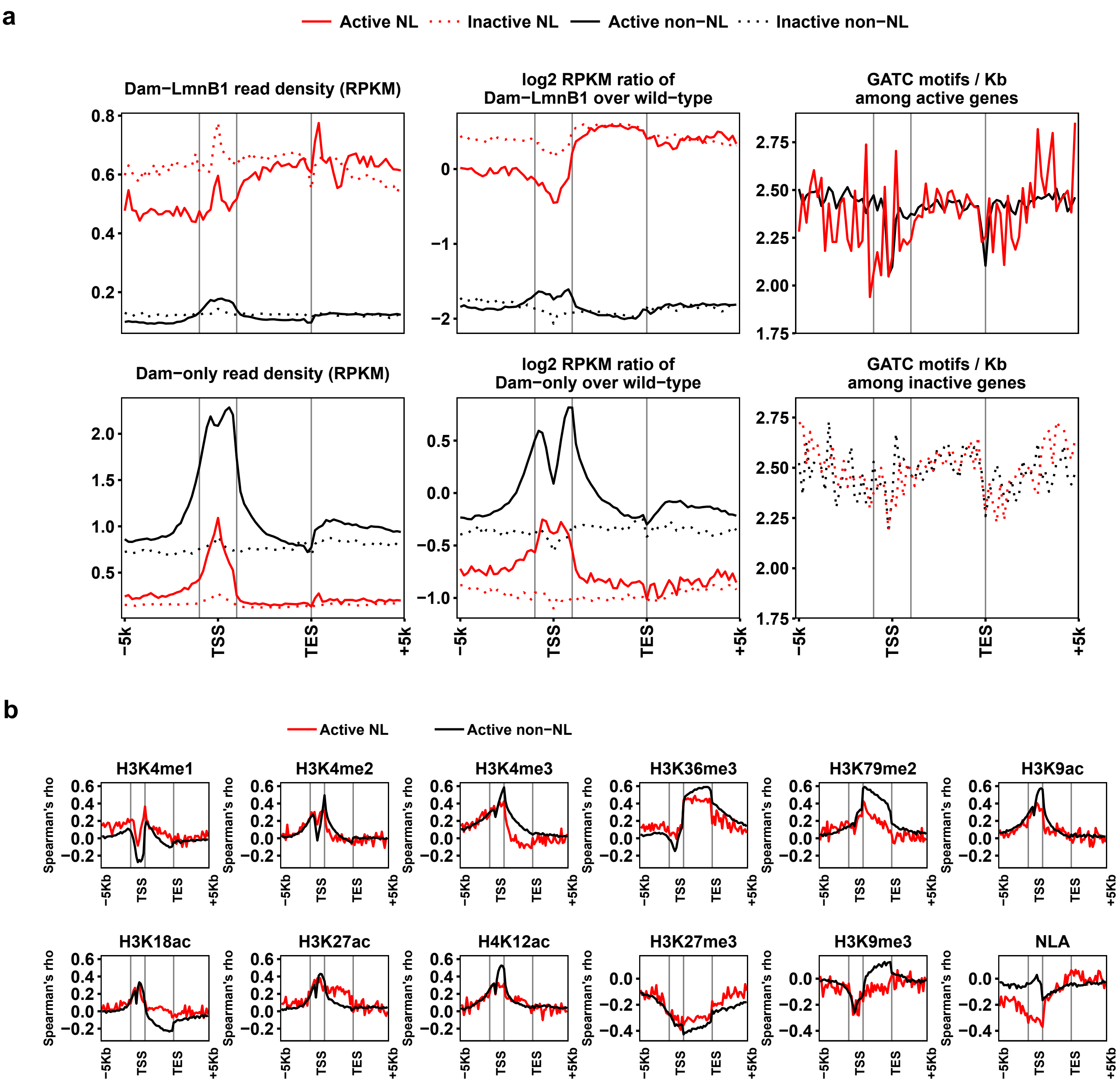

Figure S3. Plots of additional DamID-seq data in C2C12 myoblasts. (a) Plots comparing average read densities of Dam-LmnB1 and Dam-only, their read densities normalized to the wild-type genomic DNA sample and average GATC frequencies across genic regions among active/inactive NL and non-NL genes. **(b)** Comparing patterns of Spearman's correlations of gene activity relative to histone modifications and NLA across genic regions between active NL genes and active non-NL genes. Data are binned as described in Fig. 3b. Grey vertical lines from left to right indicate TSS -1 Kb, TSS +1 Kb and TES, respectively.

Figure S4 (C2C12 myoblasts)

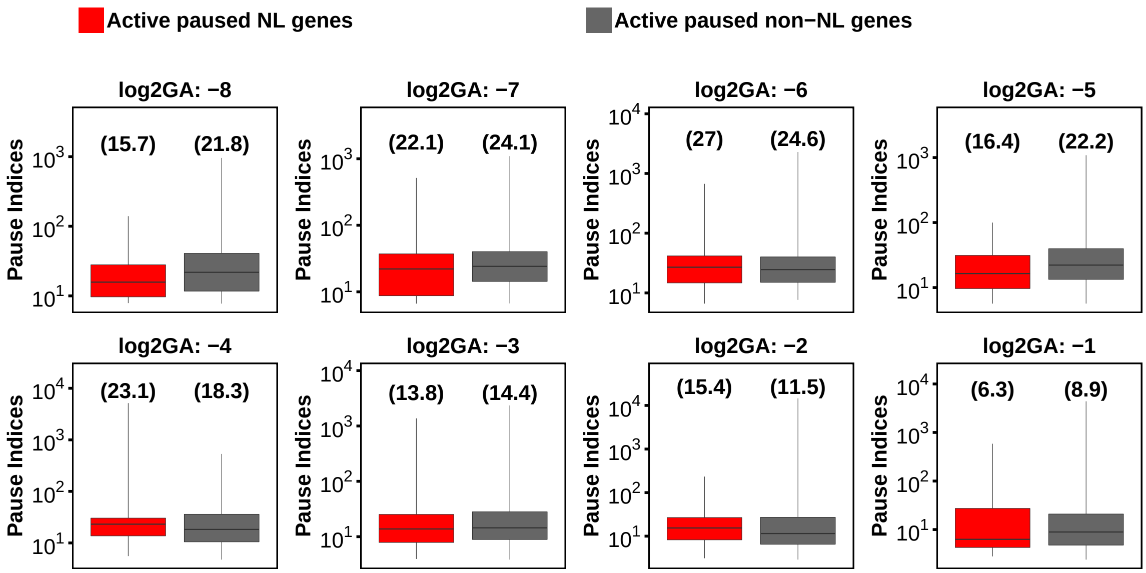

Figure S4. Box plots comparing pause indices (PIs) of active paused NL and non-NL genes grouped by gene activity in C2C12 myoblasts. Genes are grouped by rounding log2 of gene activity (Log2GA) to the nearest integer. The values of log2GA (log2RPK$_m$M) are shown above each plot. NL genes and non-NL genes show no significant differences in PI at most gene activity group (two-sided Mann-Whitney test, p-values > 0.05) except for the group with log2GA of -5 (p-value = 0.02). Only gene groups with more than 20 genes were analysed.

Figure S5 (NIH 3T3 fibroblasts)

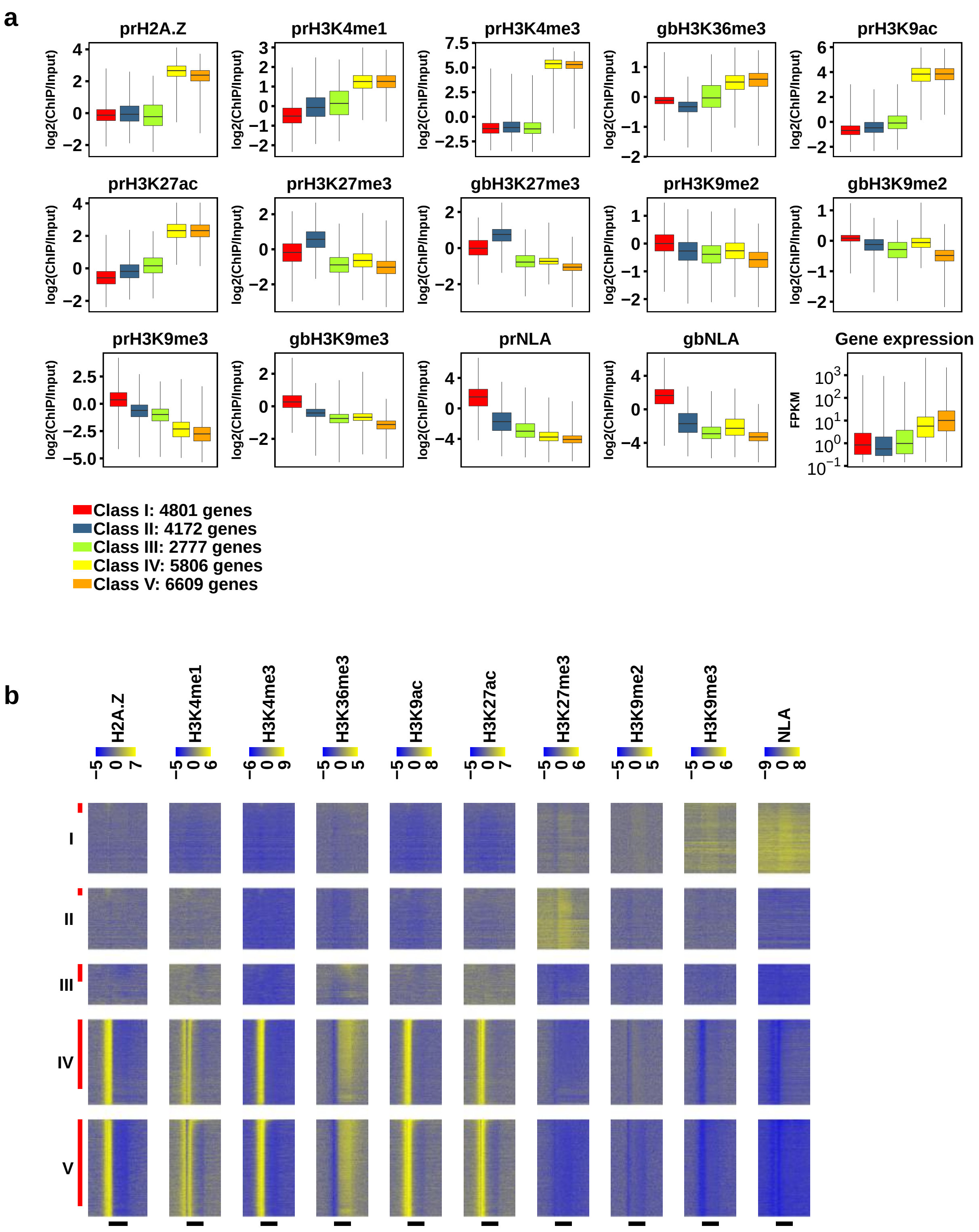

Figure S5. Clustering analysis revealed five gene classes with distinct epigenetic features in NIH 3T3 fibroblasts. (a) Box plots comparing levels of histone marks, NLA and gene expression among the identified five gene classes. The minimum and the maximum values are indicated by whisker ends and quartiles by box boundaries. **(b)** Heat maps of histone marks, histone variant H2A.Z and NLA across genic regions. Genes in each class are sorted by decreasing gene expression. Vertical red bars on the left mark expressed genes determined by RNA-seq (13.71%, 11.74%, 43.07%, 81.55% and 89.54% of Class I-V genes, respectively). Horizontal black bars at the bottom mark annotated transcribed regions from TSS to TES. Data are binned as described in Fig. 3b.

Figure S6 (C2C12 myoblasts)

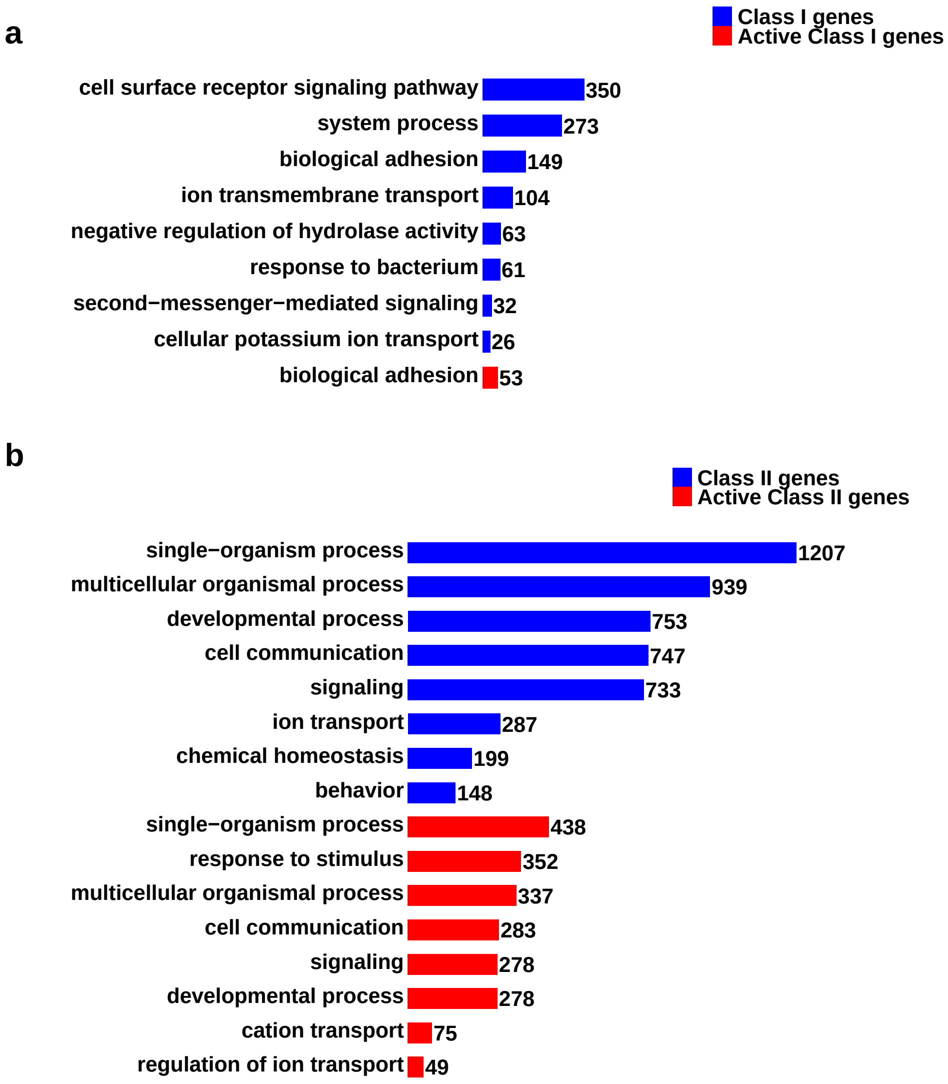

Figure S6. Gene Ontology analyses of Class I and II genes in C2C12 myoblasts.

Legends follow Fig. 2.

Figure S7 (C2C12 myoblasts)

a

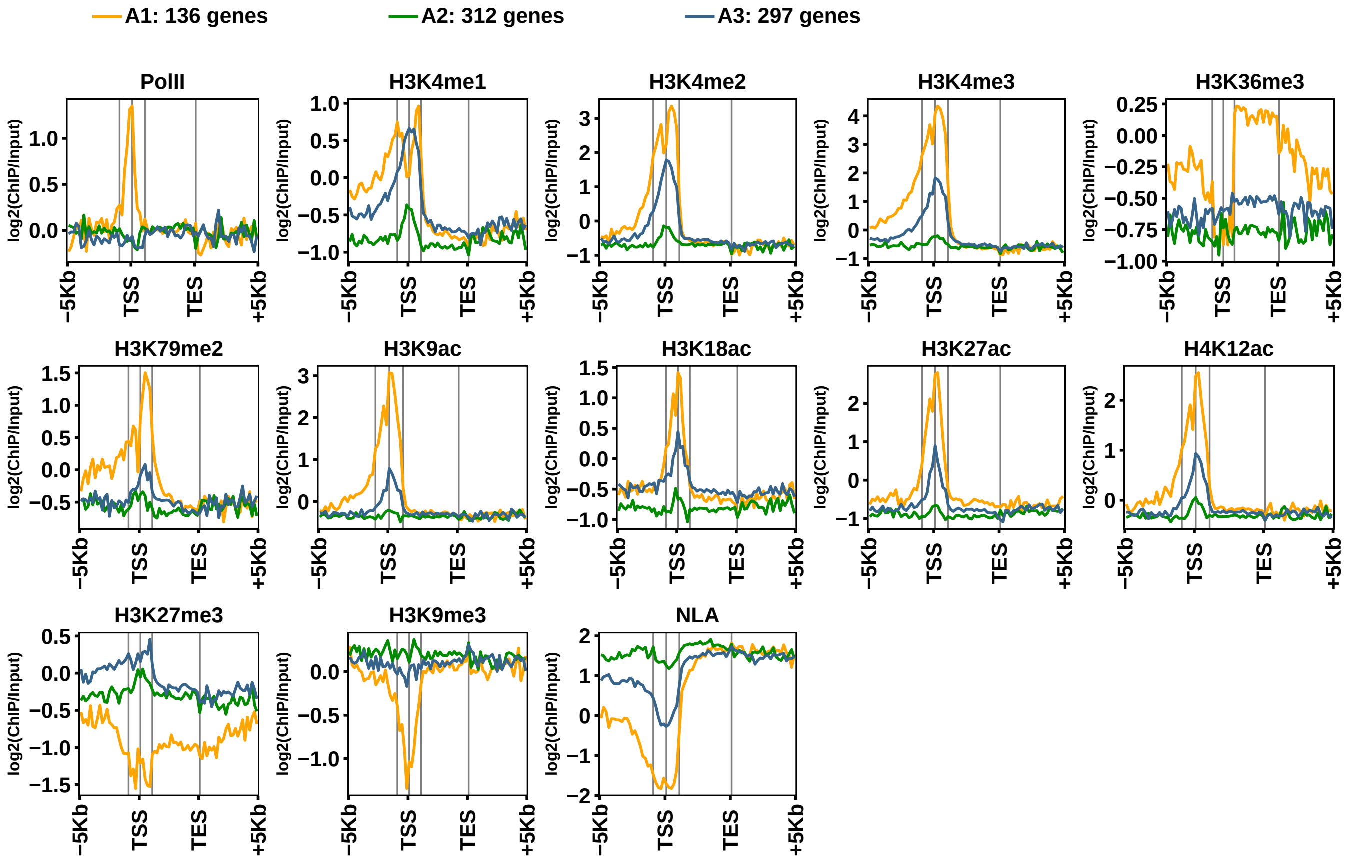

b

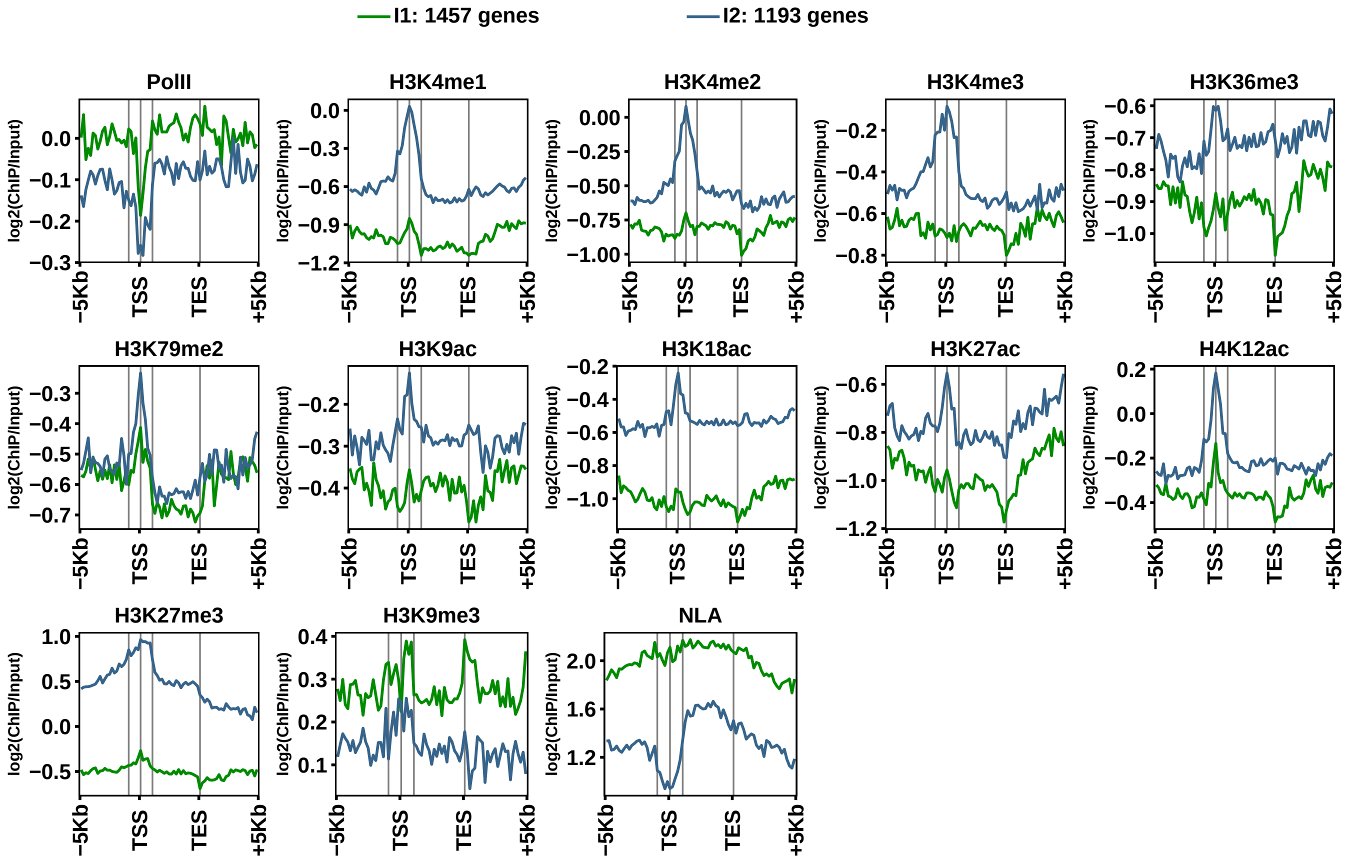

Figure S7. NL genes are differentially enriched with heterochromatic histone marks in C2C12 myoblasts. Plots displaying average levels of Pol II, histone marks and NLA across genic regions among the three groups (A1-A3) of active NL genes (a) and among the two groups (I1-I2) of inactive NL genes (b). Data are binned as described in Fig. 3b.

Figure S8 (C2C12 myoblasts)

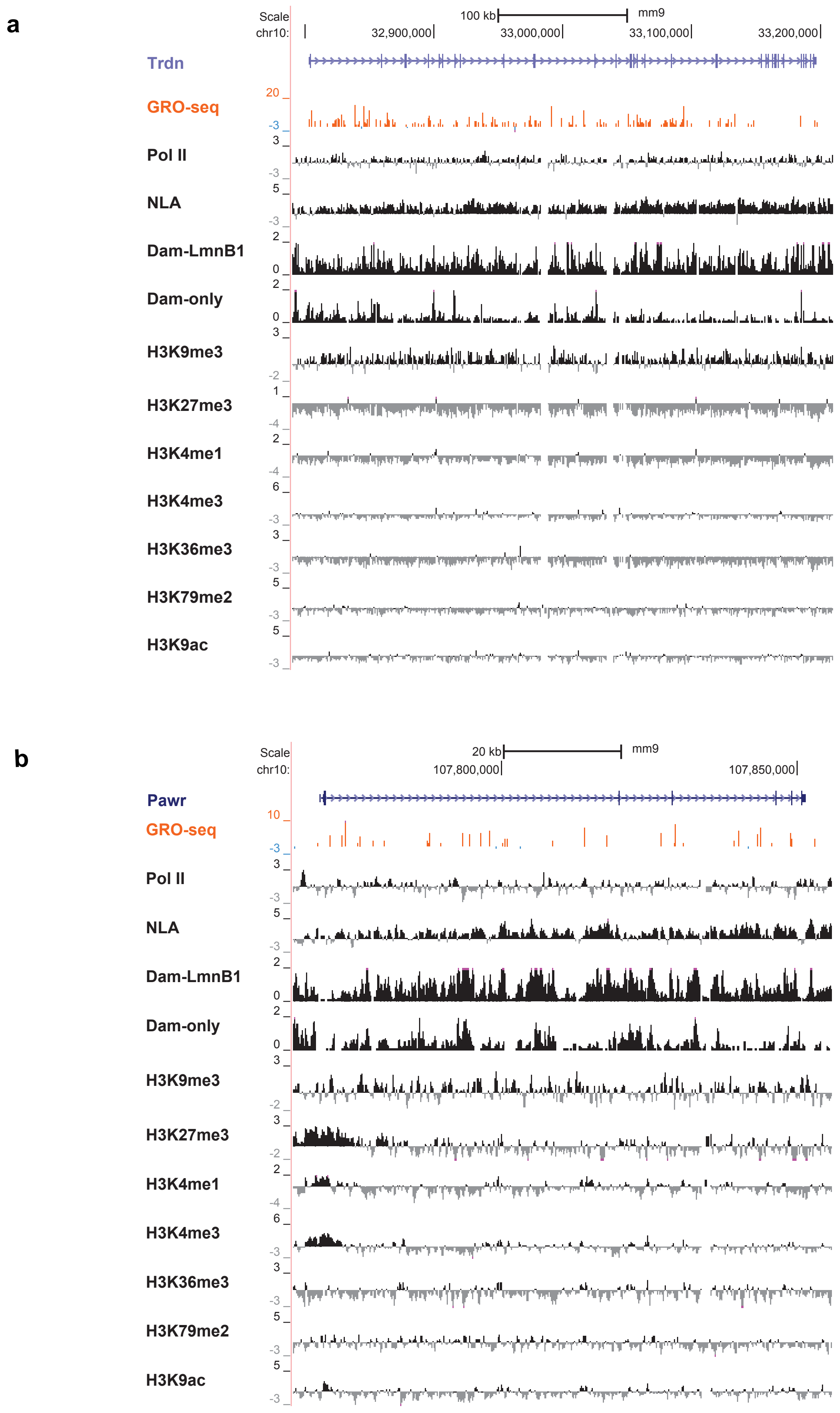

Figure S8 continued (C2C12 myoblasts)

c

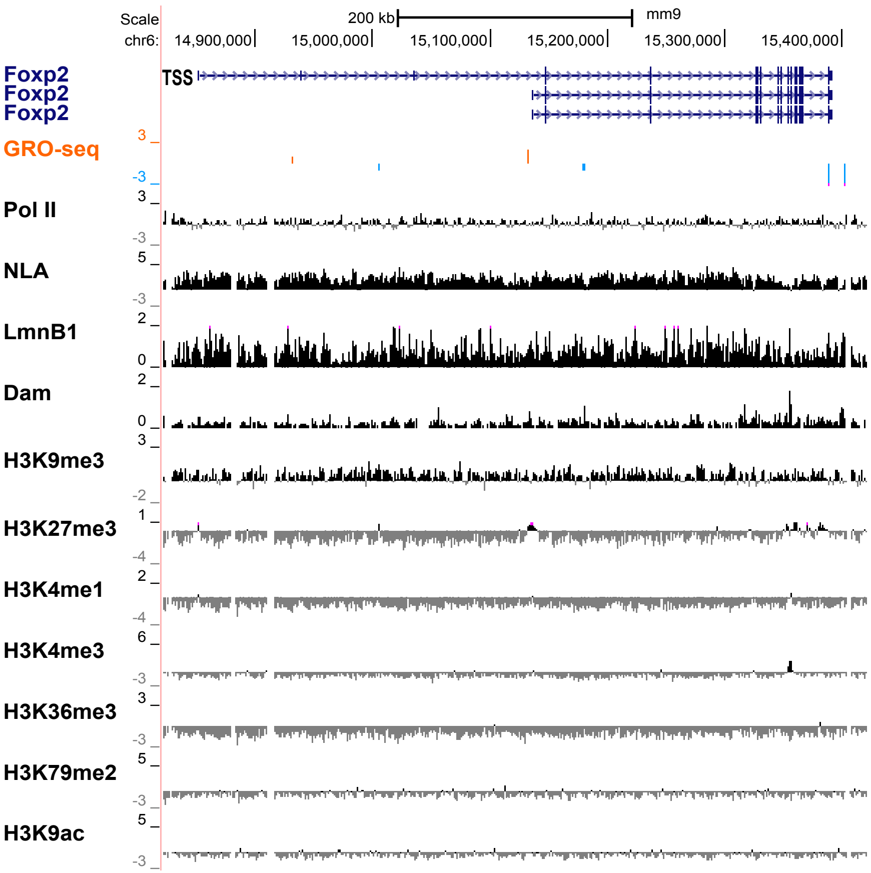

d

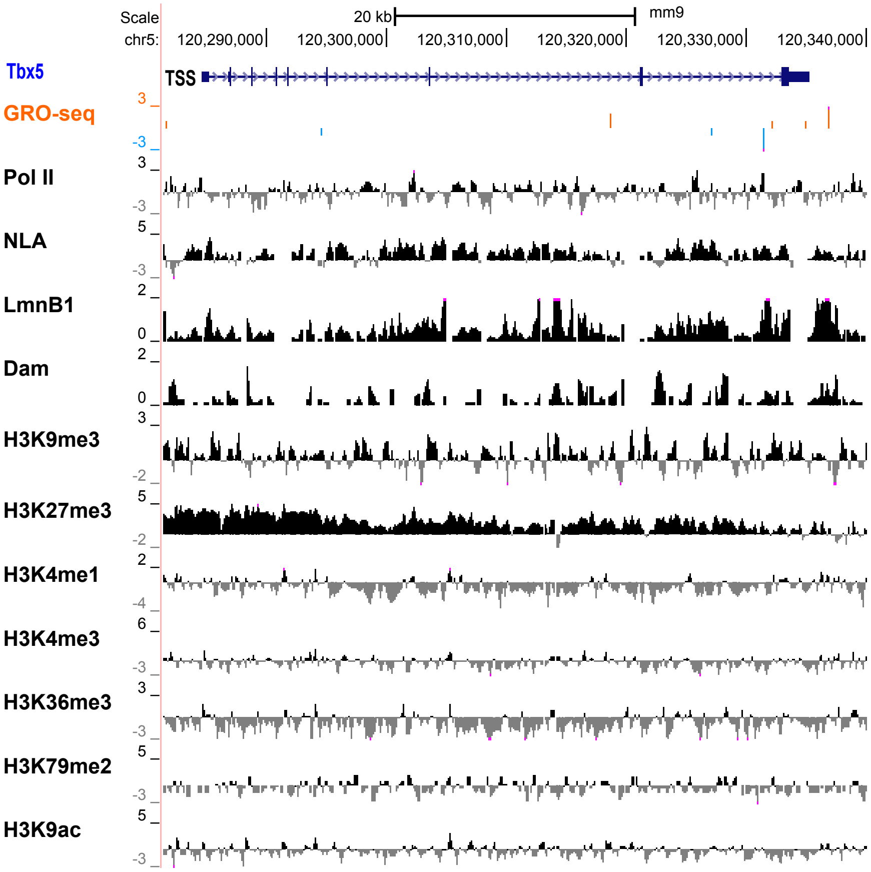

Figure S8. UCSC genome browser views of two active NL genes and two inactive NL genes in C2C12 myoblasts. (a) *Trdn* (A2 gene), **(b)** *Pawr* (A3 gene), **(c)** *Foxp2* (I1 gene), **(d)** *Tbx5* (I2 gene). Legends follow Fig. 3a.

Figure S9 (NIH 3T3 fibroblasts)

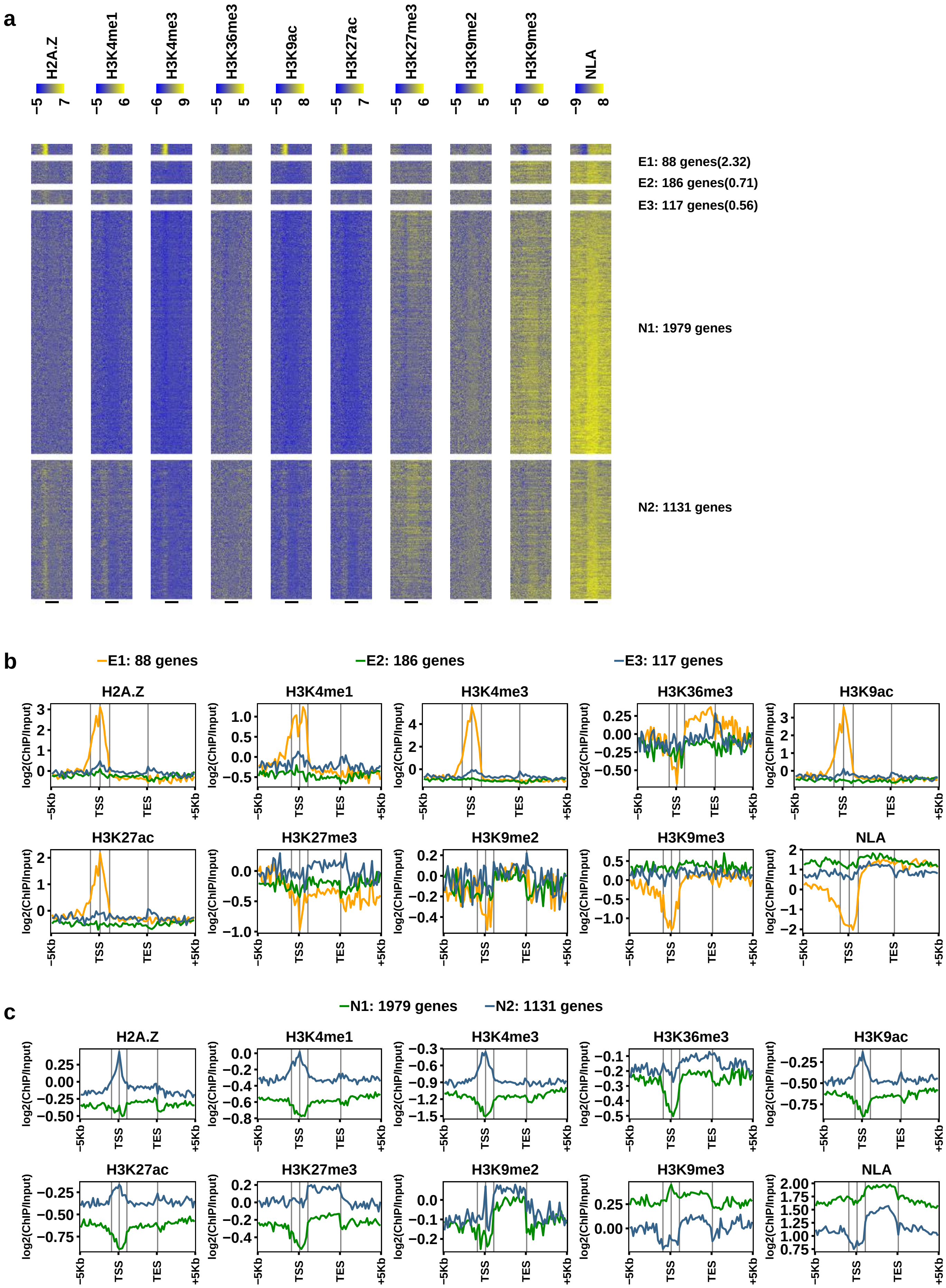

Figure S9 continued (NIH 3T3 fibroblasts)

d

TSS locations relative to LADs among NL genes of which gene bodies overlap with LADs

NL gene group	TSS within LADs	TSS outside LADs	TSS outside LADs (%)
E1	40	21	34.4%
E2	136	7	4.9%
E3	76	11	12.6%
N1	1568	73	4.4%
N2	776	86	10.0%

Figure S9. NL genes are differentially enriched with heterochromatic histone marks in NIH 3T3 fibroblasts. (a) Heat maps displaying levels of H2A.Z, histone marks and NLA across genic regions among three groups (E1-E3) of expressed NL genes and two groups (N1-N2) of non-expressed NL genes. Genes in E1-E3 are sorted by decreasing gene expression and in N1-N2 by increasing gene body NLA. Black bars at the bottom mark annotated transcribed regions from TSS to TES. Median gene expression levels of each expressed gene group are shown in parentheses. **(b-c)** Plots displaying average levels of H2A.Z, histone marks and NLA across genic regions among the three groups (E1-E3) of expressed NL genes (b) and among the two groups (N1-N2) of non-expressed NL genes (c). In panels b and c, data are binned as described in Fig. 3b. **(d)** TSS locations relative to LADs among genes of which gene bodies overlap with LADs. E1 genes have a higher fraction of TSSs outside LADs than the other groups (Fisher' exact test, p-values < 0.002).

Figure S10 (C2C12 myoblasts, dREG enhancers)

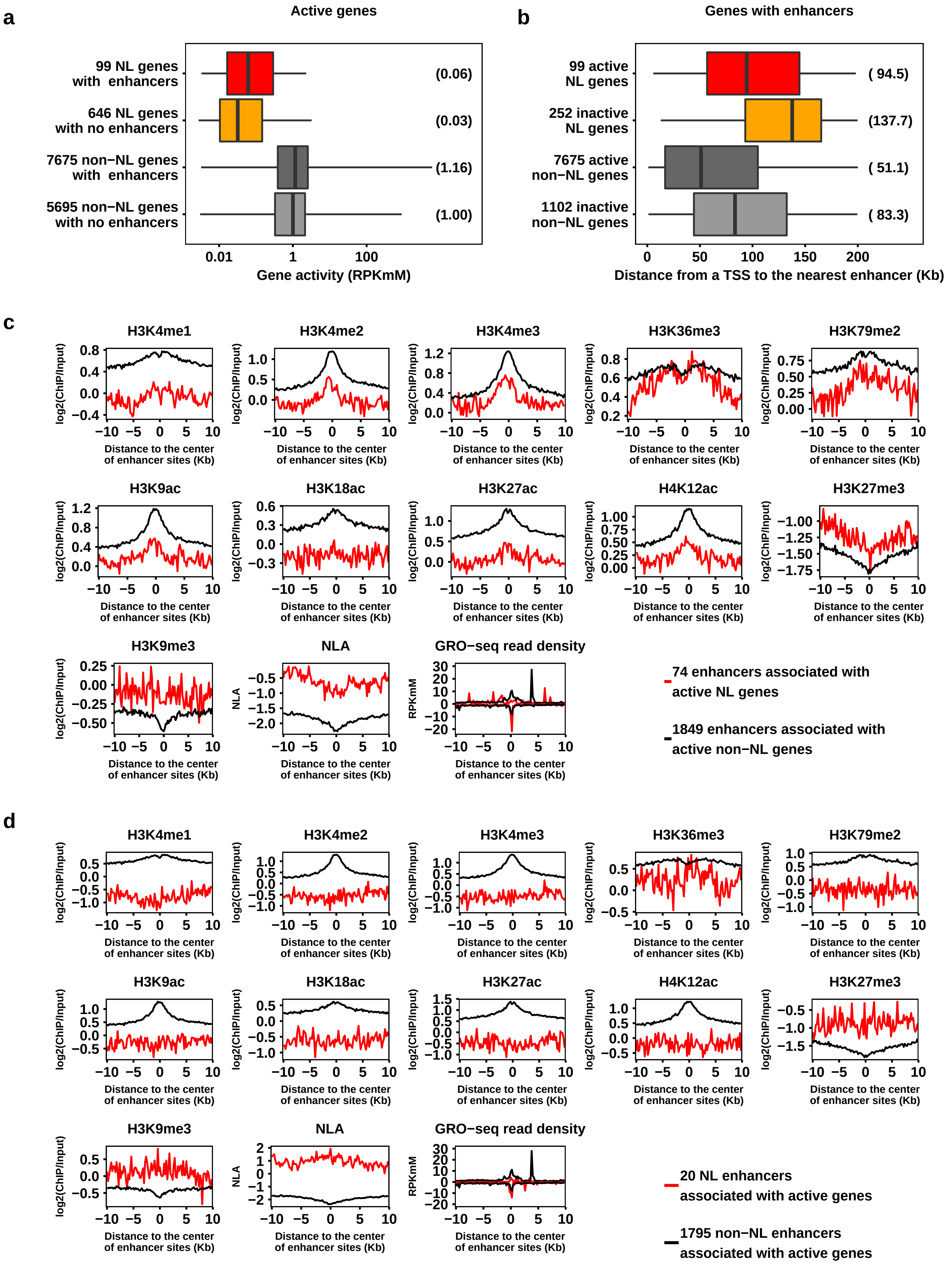

Figure S10. Analysis of dREG enhancers. (a) Box plots comparing gene activities of active NL genes and active non-NL genes with or without an associated dREG enhancer. Mann-Whitney tests revealed significant differences in all pairs of gene groups (p-values < 0.005). **(b)** Box plots comparing distances of each TSS to the nearest dREG enhancer among active and inactive NL/non-NL genes. Mann-Whitney tests revealed significant differences in all pairs of gene groups (p-value of 0.04 when comparing active NL genes and inactive non-NL genes and p-values < 0.0001 for all other pairwise comparisons). **(c)** Plots comparing average levels of histone marks, NLA and GRO-seq read density (in the regions of enhancer centre ± 10 Kb) for dREG enhancers associated with active NL genes (red) and dREG enhancers associated with active non-NL genes (black). **(d)** Plots comparing average levels of histone marks, NLA and GRO-seq read density (in the regions of enhancer centre ± 10 Kb) for NL dREG enhancers associated with active genes (red) and non-NL dREG enhancers associated with active genes (black). Data of an enhancer were computed only once when the enhancer is associated with multiple genes in the same group.

Figure S11 (C2C12 myoblasts, Blum enhancers)

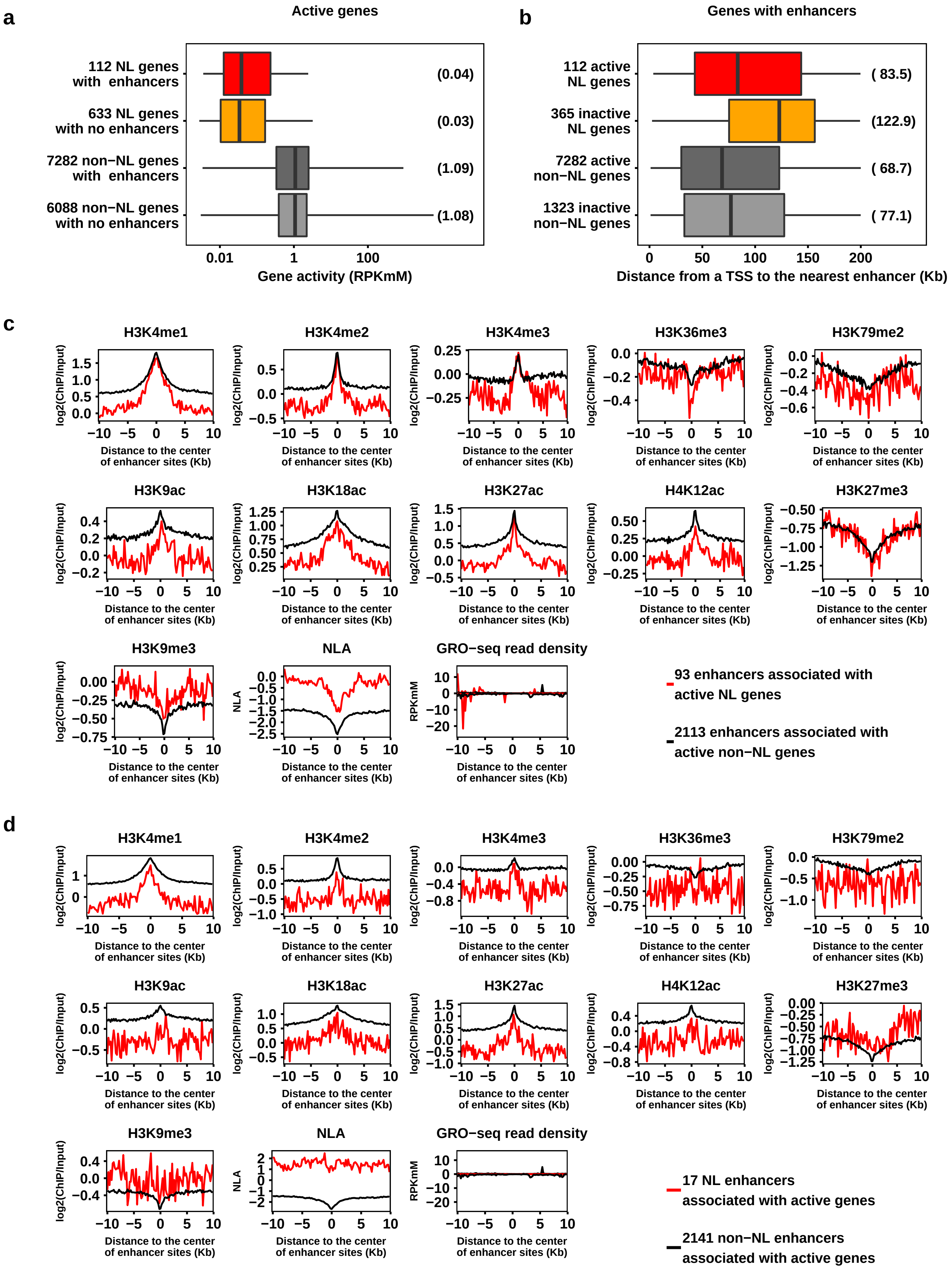

Figure S11. Analysis of Blum enhancers. (a) Box plots comparing gene activities of active NL genes and active non-NL genes with or without an identified Blum enhancer [2]. Mann-Whitney tests revealed no differences in gene activity between NL genes (or non-NL genes) with and without an identified Blum enhancer (p-value = 0.13 for both cases), but significant differences in gene activity between all other pairs of gene groups (p-values < 1e-37). **(b)** Box plots comparing distances of each TSS to its nearest Blum enhancer distances among active and inactive NL/non-NL genes. Mann-Whitney tests revealed significant differences in all pairs of gene groups (p-value of 0.02 when comparing active NL genes and inactive non-NL genes and p-values < 0.005 for all other pairwise comparisons). **(c)** Plots comparing average levels of histone marks, NLA and GRO-seq read density (in the regions of enhancer centre ± 10 Kb) of Blum enhancers associated with active NL genes (red) and Blum enhancers associated with active non-NL genes (black). **(d)** Plots comparing average levels of histone marks, NLA and GRO-seq read density (in the regions of enhancer centre ± 10 Kb) of NL Blum enhancers associated with active genes (red) and non-NL Blum enhancers associated with active genes (black). Data of an enhancer were computed only once when the enhancer is associated with multiple genes in the same group.

Figure S12 (C2C12 myoblasts, super enhancers)

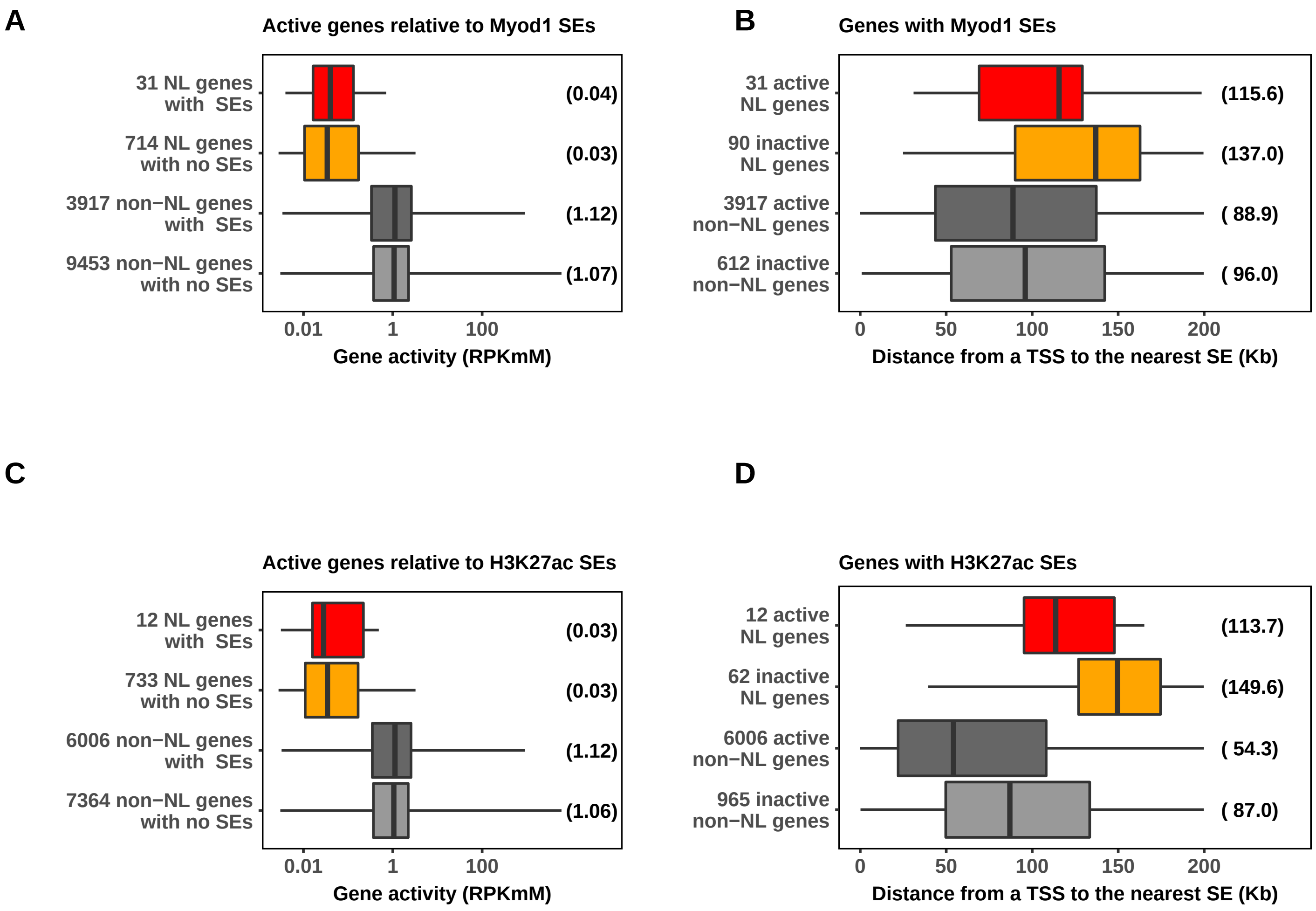

Figure S12. Analysis of SEs. (a) Box plot comparing gene activities of active NL genes and active non-NL genes with or without an identified MyoD1 SE. Mann-Whitney tests revealed no difference in gene activity between NL genes with and without an identified MyoD1 SE (p-value = 0.30), marginal difference in gene activity between non-NL genes with and without an identified MyoD1 SE (p-value = 0.04), but significant differences in gene activity between all other pairs of gene groups (p-values < 4e-13). **(b)** Box plot comparing distances from each TSS to its nearest MyoD1 SE boundary among active and inactive NL/non-NL genes. Mann-Whitney tests revealed no difference between active NL genes and inactive non-NL genes (p-value = 0.11), marginal differences when comparing active NL genes versus inactive NL genes or versus active non-NL genes (p-values of 0.02 and 0.04, respectively), and significant differences in all other pairs (p-values < 0.004). **(c)** Box plot comparing gene activities of active NL genes and active non-NL genes with or without an identified H3K27ac SE. Mann-Whitney tests revealed no difference in gene activity between NL genes with and without an identified H3K27ac SE (p-value = 0.51), but significant differences in gene activity between all other pairs of gene groups (p-value of 0.0009 for two non-NL gene groups and p-values < 2e-6 for others). **(d)** Box plot comparing distances from each TSS to its nearest H3K27ac SE boundary among active and inactive NL/non-NL genes. Mann-Whitney tests revealed significant differences in all pairs of gene groups (p-values < 0.003) except between active NL genes and inactive non-NL genes (p-value = 0.07).

Supplementary Tables

Table S1 - NL genes in C2C12 myoblasts. Inactive genes and non-paused genes are indicated by "NA" in columns "Gene activity" and "Pause index", respectively.

Table S2 - NL genes in NIH 3T3 fibroblasts. Non-expressed genes are indicated by "NA" in column "Gene expression".

Table S3 – Distributions of NL genes over LADs in NIH 3T3 fibroblasts.

Table S4 - GO analyses in C2C12 myoblasts. Listed Biological Process categories are enriched with FDRs < 0.05.

Table S5 - NL genes grouped by transcription status and promoter NL-association. For the published DamID-microarray data [1], we estimated prNLA by averaging log2(Dam-Lamin B1/Dam) of probes overlapping the promoter region. Note that some RefSeq genes do not have corresponding probes on the microarray. Fisher's exact tests revealed that prNLA is more likely to be negative among active/expressed NL genes than inactive/non-expressed NL genes (p-values = 1.3e-104, 2.4e-28 and 3.8e-7 for C2C12 DamID-seq [3], 3T3 DamID-seq [3] and 3T3 DamID-microarray data [1], respectively).

Table S6 – Statistics on NLA, histone modifications and gene activities of five gene classes identified in C2C12 myoblasts and in NIH 3T3 fibroblasts. Histone mark levels were measured by log2 of ChIP-Input enrichment.

Table S7 - Statistics on NLA, histone modifications and gene activities of NL gene subgroups identified in C2C12 myoblasts and in NIH 3T3 fibroblasts. Histone mark levels were measured by log2 of ChIP-Input enrichment.

Table S8 - Putative enhancer sites detected by dREG using GRO-seq data in C2C12 myoblasts.

Table S9 - Genes grouped by transcription status, gene body NLA and the nearest enhancers. As one enhancer may be the nearest enhancer of multiple genes, both the gene number and the associated unique enhancer number in each group are listed and separated by a slash.

Table S10 - Putative super enhancer sites detected by ROSE using MyoD1 and H3K27ac ChIP-seq data in C2C12 myoblasts.

Table S11 - Genes grouped by transcription status, gene body NLA and the nearest SEs. As one SE may be the nearest SE of multiple genes, both the gene number and the associated unique SE number in each group are listed and separated by a slash.

Table S12 -- Analysed high throughput sequencing data and microarray data.

References

1 Peric-Hupkes, D. *et al.* Molecular maps of the reorganization of genome-nuclear lamina interactions during differentiation. *Mol Cell* **38**, 603-613, doi:10.1016/j.molcel.2010.03.016 (2010).

2 Blum, R., Vethantham, V., Bowman, C., Rudnicki, M. & Dynlacht, B. D. Genome-wide identification of enhancers in skeletal muscle: the role of MyoD1. *Genes Dev* **26**, 2763-2779, doi:10.1101/gad.200113.112 (2012).

3 Wu, F. & Yao, J. Spatial compartmentalization at the nuclear periphery characterized by genome-wide mapping. *BMC Genomics* **14**, 591, doi:10.1186/1471-2164-14-591 (2013).
